# Endothelial insulin-like growth factor-1 signalling regulates vascular barrier function and atherogenesis

**DOI:** 10.1093/cvr/cvaf055

**Published:** 2025-04-02

**Authors:** Michael Drozd, Alexander-Francisco Bruns, Nadira Y Yuldasheva, Azhar Maqbool, Hema Viswambharan, Anna Skromna, Natallia Makava, Chew W Cheng, Piruthivi Sukumar, Lauren Eades, Andrew M N Walker, Kathryn J Griffin, Stacey Galloway, Nicole T Watt, Natalie Haywood, Victoria Palin, Nele Warmke, Helen Imrie, Katherine Bridge, David J Beech, Stephen B Wheatcroft, Mark T Kearney, Richard M Cubbon

**Affiliations:** Leeds Institute of Cardiovascular and Metabolic Medicine, LIGHT Laboratories, Clarendon Way, University of Leeds, Leeds LS2 9JT, UK; Leeds Institute of Cardiovascular and Metabolic Medicine, LIGHT Laboratories, Clarendon Way, University of Leeds, Leeds LS2 9JT, UK; Leeds Institute of Cardiovascular and Metabolic Medicine, LIGHT Laboratories, Clarendon Way, University of Leeds, Leeds LS2 9JT, UK; Leeds Institute of Cardiovascular and Metabolic Medicine, LIGHT Laboratories, Clarendon Way, University of Leeds, Leeds LS2 9JT, UK; Leeds Institute of Cardiovascular and Metabolic Medicine, LIGHT Laboratories, Clarendon Way, University of Leeds, Leeds LS2 9JT, UK; Leeds Institute of Cardiovascular and Metabolic Medicine, LIGHT Laboratories, Clarendon Way, University of Leeds, Leeds LS2 9JT, UK; Leeds Institute of Cardiovascular and Metabolic Medicine, LIGHT Laboratories, Clarendon Way, University of Leeds, Leeds LS2 9JT, UK; Leeds Institute of Cardiovascular and Metabolic Medicine, LIGHT Laboratories, Clarendon Way, University of Leeds, Leeds LS2 9JT, UK; Leeds Institute of Cardiovascular and Metabolic Medicine, LIGHT Laboratories, Clarendon Way, University of Leeds, Leeds LS2 9JT, UK; Leeds Institute of Cardiovascular and Metabolic Medicine, LIGHT Laboratories, Clarendon Way, University of Leeds, Leeds LS2 9JT, UK; Leeds Institute of Cardiovascular and Metabolic Medicine, LIGHT Laboratories, Clarendon Way, University of Leeds, Leeds LS2 9JT, UK; Leeds Institute of Cardiovascular and Metabolic Medicine, LIGHT Laboratories, Clarendon Way, University of Leeds, Leeds LS2 9JT, UK; Leeds Institute of Cardiovascular and Metabolic Medicine, LIGHT Laboratories, Clarendon Way, University of Leeds, Leeds LS2 9JT, UK; Leeds Institute of Cardiovascular and Metabolic Medicine, LIGHT Laboratories, Clarendon Way, University of Leeds, Leeds LS2 9JT, UK; Leeds Institute of Cardiovascular and Metabolic Medicine, LIGHT Laboratories, Clarendon Way, University of Leeds, Leeds LS2 9JT, UK; Leeds Institute of Cardiovascular and Metabolic Medicine, LIGHT Laboratories, Clarendon Way, University of Leeds, Leeds LS2 9JT, UK; Leeds Institute of Cardiovascular and Metabolic Medicine, LIGHT Laboratories, Clarendon Way, University of Leeds, Leeds LS2 9JT, UK; Leeds Institute of Cardiovascular and Metabolic Medicine, LIGHT Laboratories, Clarendon Way, University of Leeds, Leeds LS2 9JT, UK; Leeds Institute of Cardiovascular and Metabolic Medicine, LIGHT Laboratories, Clarendon Way, University of Leeds, Leeds LS2 9JT, UK; Leeds Institute of Cardiovascular and Metabolic Medicine, LIGHT Laboratories, Clarendon Way, University of Leeds, Leeds LS2 9JT, UK; Leeds Institute of Cardiovascular and Metabolic Medicine, LIGHT Laboratories, Clarendon Way, University of Leeds, Leeds LS2 9JT, UK; Leeds Institute of Cardiovascular and Metabolic Medicine, LIGHT Laboratories, Clarendon Way, University of Leeds, Leeds LS2 9JT, UK; Leeds Institute of Cardiovascular and Metabolic Medicine, LIGHT Laboratories, Clarendon Way, University of Leeds, Leeds LS2 9JT, UK

**Keywords:** Insulin-like growth factor-1 receptor, Endothelial, Vascular permeability, Atherosclerosis

## Abstract

**Aims:**

Progressive deposition of cholesterol in the arterial wall characterizes atherosclerosis, which underpins most cases of myocardial infarction and stroke. Insulin-like growth factor-1 (IGF-1) is a hormone that regulates systemic growth and metabolism and possesses anti-atherosclerotic properties. We asked whether endothelial-restricted augmentation of IGF-1 signalling is sufficient to suppress atherogenesis.

**Methods and results:**

We generated mice with endothelial-restricted over-expression of human wild-type (WT) IGF-1R (hIGFREO/ApoE^−/−^) or a signalling-defective K1003R mutant human IGF-1R (mIGFREO/ApoE^−/−^) and compared them with their respective ApoE^−/−^ littermates. hIGFREO/ApoE^−/−^ had less atherosclerosis, circulating leucocytes, arterial cholesterol uptake, and vascular leakage in multiple organs, whereas mIGFREO/ApoE^−/−^ did not exhibit these phenomena. Over-expressing WT IGF-1R in human umbilical vein endothelial cells (HUVECs) altered the localization of tight junction proteins and reduced paracellular leakage across their monolayers, whilst over-expression of K1003R IGF-1R did not have these effects. Moreover, only over-expression of WT IGF-1R reduced HUVEC internalization of cholesterol-rich low-density lipoprotein particles and increased their association of these particles with clathrin, but not caveolin-1, implicating it in vesicular uptake of lipoproteins. Endothelial over-expression of WT vs. K1003R IGF-1R also reduced expression of YAP/TAZ target genes and nuclear localization of TAZ, which may be relevant to its impact on vascular barrier and atherogenesis.

**Conclusion:**

Endothelial IGF-1 signalling modulates both para- and transcellular vascular barrier function. Beyond reducing atherosclerosis, this could have relevance to many diseases associated with abnormal vascular permeability.

## Graphical Abstract

**Graphical Abstract:**
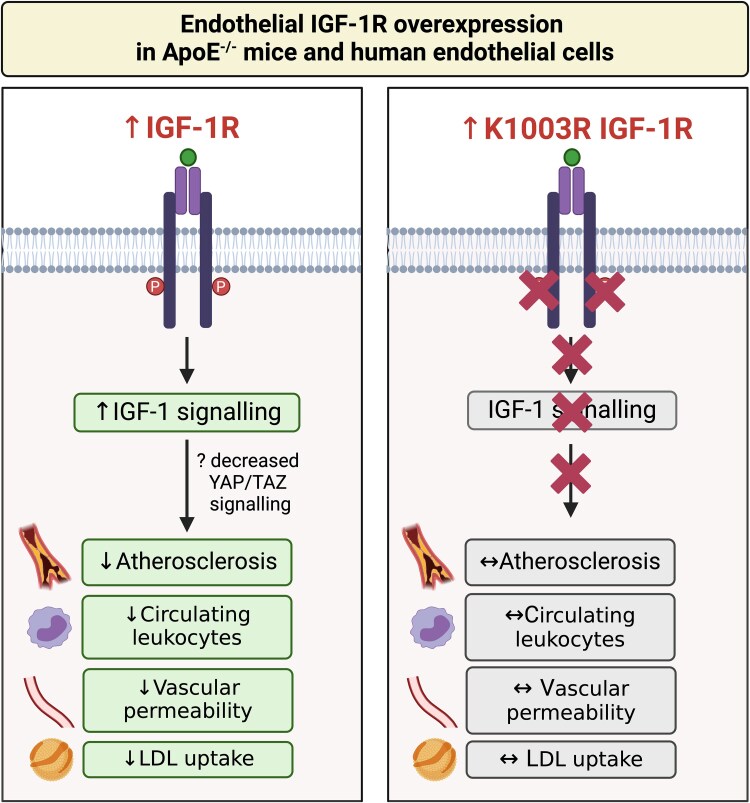

**Time of primary review: 54 days**

**See the editorial comment for this article ‘Endothelial IGF-1 signalling: a conductor of vascular barrier function and low-density lipoprotein trafficking in atherogenesis Endothelial IGF-1 signalling: a conductor of vascular barrier function and low-density lipoprotein trafficking in atherogenesis’, by C. Liu and J. Kalucka, https://doi.org/10.1093/cvr/cvaf082.**

## 1. Introduction

Atherosclerosis is the leading cause of death and disability worldwide^1^ and is likely to remain a major public health problem as the projected global epidemic of Type 2 diabetes mellitus (T2DM) unfolds during the first half of this century.^2^ Dysfunction of the vascular endothelial lining is thought to initiate this process, permitting cholesterol-rich lipoproteins to transit from the circulation into the arterial wall, forming atherosclerotic plaques.^3^ Pathological, experimental, and therapeutic evidence now strongly support the notion that atherosclerosis is a chronic inflammatory disorder.^3,4^ This inflammation is initiated by progressive accumulation of cholesterol-rich lipoproteins within the arterial wall, forming necrotic cores isolated from the bloodstream by a protective ‘fibrous cap’. Passage of circulating lipoproteins into the arterial wall requires transit across the vascular endothelial lining, either via gaps between adjacent endothelial cells (paracellular) or by active transport by endothelial cells (transcellular).^5^

Insulin-like growth factor-1 (IGF-1) is an endocrine and autocrine/paracrine growth factor that regulates cell survival, growth, and metabolism.^6^ Its cognate receptor tyrosine kinase (IGF-1R) is widely expressed in endothelial cells,^7^ especially brain endothelial cells,^8–10^ where its role is poorly explored. However, it is notable that the blood–brain barrier endothelium is unusually resistant to paracellular leakage and transcellular cholesterol transport. Low concentrations of circulating IGF-1 are implicated in the development of human atherosclerosis.^11–13^ IGF-1R expression is reduced in advanced human atherosclerotic plaque^14^ and in the aorta of obese mice.^15^ Consistent with an anti-atherosclerotic action, studies of cultured endothelial cells have shown IGF-1 to have antioxidant effects,^16^ to increase production of the anti-atherosclerotic signalling radical nitric oxide (NO),^17^ and to protect against reactive oxygen species (ROS)-mediated endothelial cell senescence and apoptosis.^18^ Atherosclerosis prone apolipoprotein E knockout (ApoE^−/−^) mice deficient in IGF-1 develop accelerated atherosclerosis,^19^ whilst administration of IGF-1 to ApoE^−/−^ mice can slow the development of atherosclerosis.^20^

Despite abundant expression of IGF-1R in endothelial cells, data on the role of endothelial IGF-1/IGF-1R in vascular biology remain limited. Some studies suggest that augmenting endothelial IGF-1 signalling limits paracellular vascular leakage by increasing the expression or activation of proteins forming endothelial cell–cell junctions.^21,22^ However, as most cholesterol accumulation in the arterial wall is presumed to be via transcellular transport,^5^ the relevance of IGF-1 to atherosclerosis via endothelial junctions alone is unclear. IGF-1 receptor (IGF-1R) expression is enriched in brain endothelial cells.^8–10^ Notably, the brain vasculature is unusually resistant to cholesterol transit, both via passive leakage between endothelial cells *and* active trans-endothelial transit.^23,24^ Here, we show that ApoE^−/−^ mice with transgenic over-expression of human IGF-1R in the endothelium (hIGFREO/ApoE^−/−^) have reduced atherosclerosis with reduced paracellular vascular leakage *and* cellular cholesterol uptake.

## 2. Methods

### 2.1 Animals and animal procedures

Mice were maintained in a temperature- and humidity-controlled environment with a 12 h light-dark cycle. Genotyping was performed using polymerase chain reaction (PCR) amplification of ear notch genomic DNA. hIGFREO/ApoE^−/−^ were generated by crossing ApoE^−/−^ mice with human IGF-1 receptor endothelium over-expressing mice (hIGFREO).^25^ mIGFREO/ApoE^−/−^ were generated by crossing ApoE^−/−^ mice with K1003R mutant human IGF-1 receptor endothelium over-expressing mice (mIGFREO).^26^ Male mice were studied in all experiments. This work was conducted with local institutional approval and in accordance with guidelines from Directive 2010/63/EU of the European Parliament on the protection of animals used for scientific purposes under United Kingdom Home Office project license P144DD0D6.

### 2.2 *In vivo* assessment of glucose and lipid homeostasis

*In vivo* metabolic testing was performed, as previously described.^27,28^ For glucose tolerance tests, mice were fasted for 16 h, followed by intraperitoneal (IP) injection of 1 g/kg glucose. For insulin tolerance tests, mice were fasted for 4 h, followed by IP injection of 0.75 units/kg insulin (Actrapid; NovoNordisk, Bagsvaerd, Denmark), after which blood glucose was determined at 30 min intervals by tail vein sampling using a portable meter (Accu-chek Aviva; Roche Diagnostics, Burgess Hill, UK). Plasma insulin and IGF-1 were measured using ultrasensitive mouse ELISA kits (CrystalChem, Downers Grove, IL, USA and R&D Systems, Minneapolis, MN, USA), as previously described.^15^ Triglycerides and cholesterol were measured in fasting plasma using colorimetric assays (Abcam, Cambridge, UK) as described.^28^

### 2.3 *In vivo* blood pressure measurement

Systolic blood pressure was measured using tail-cuff plethysmography in conscious mice.^29,30^ Mice were pre-warmed for 10 min in a thermostatically controlled restrainer (CODA2 System, XBP1000; Kent Scientific, Torrington, CT). Three training sessions were performed during the week before measurements were taken. The mean of at least five separate recordings on three occasions was taken to calculate mean systolic blood pressure.

### 2.4 Studies of vasomotor function in aortic rings

Aortic vasomotor function was assessed, as previously described.^27–31^ A cumulative dose response to the constrictor phenylephrine (1 nmol/L to 10 μmol/L) was first performed followed by relaxation responses to the endothelium-dependent vasodilator acetylcholine (Ach; 1 nmol/L–10 µmol/L) and endothelium-independent vasodilator sodium nitroprusside (SNP; 0.1 nmol/L–1 µmol/L), responses are expressed as % change in pre-constricted tension. Basal NO in response to isometric tension was assessed as the constrictor response to the non-selective NO synthase (NOS) inhibitor L-NMMA (0.1 mM) in aortic segments maximally pre-constricted with phenylephrine. The effects of the superoxide dismutase/catalase mimetic MnTmPyP (10 μmol/L for 30 min; Merck, Gillingham, UK) on aortic relaxation were examined, as previously reported.^28^

### 2.5 NO synthase activity

The effect of insulin and IGF-1 on eNOS activity in aorta was determined by conversion of [^14^C]-L-arginine to [^14^C]-L-citrulline as we described.^28^ Aortic segments were incubated at 37°C for 20 min in HEPES (4-(2-hydroxyethyl)-1-piperazineethanesulfonic acid) buffer pH 7.4 (in mmol/L): 10 HEPES, 145 NaCl, 5 KCl, 1 MgSO_4_, 10 glucose, 1.5 CaCl_2_ containing 0.25% bovine serum albumin (BSA). L-[^14^C] arginine, 0.5 μCi/mL, was then added for 5 min and tissues stimulated with insulin (100 nmol/L) or IGF-1 (100 nmol/L), 30 min before the reaction was stopped with cold phosphate-buffered saline (PBS), containing 5 mmol/L L-arginine and 4 mmol/L ethylenediaminetetraacetic acid (EDTA) after which tissue was denatured in 95% ethanol. After evaporation, the soluble cellular components were dissolved in 20 mmol/L HEPES-Na^+^ (pH 5.5), and applied to a well-equilibrated DOWEX (Na^+^ form), column. The L-[^14^C] citrulline content of the eluate was quantified by liquid scintillation counting and normalized against total cellular protein.

### 2.6 Quantification of atherosclerosis

After being fed a western diet for 12 weeks, mice were anaesthetized with inhaled 4% isoflurane before euthanasia with terminal exsanguination, followed by perfusion with 4% paraformaldehyde, as described.^28,30,32^ The heart was removed to study the aortic sinus, and the thoraco-abdominal aorta was used *en face* quantification of plaque.^28,30,32^ For *en face* analysis, aortas were cut longitudinally, stained with oil-red O, and photographed with an Olympus SX61 microscope. Per cent plaque area was measured using Image Pro Express software (Media Cybernetics, Rockville, MD, USA). Aortic sinus specimens were embedded in paraffin or optimum cutting temperature (OCT) compound. Sections were cut at 3 or 6 µm thickness for paraffin-embedded or OCT-embedded tissue, respectively, at the level of the aortic valve cusps. Paraffin sections were stained with Miller Van-Gieson, imaged with an Olympus B41 microscope, and plaque area quantified, as previously reported.^32^ Cryosections were incubated overnight at 4°C with rat monoclonal antibody against the macrophage marker F4/80 (Abcam, ab6640) in 1% BSA in PBS with 5% goat serum, followed by an Alexa Fluor 647–conjugated secondary antibody. Sections were mounted with Vectashield aqueous mountant (Vector Labs, Newark, CA) containing DAPI (4',6-diamidino-2-phenylindole) to reveal nuclei. Imaging was performed with a Zeiss LSM880 confocal microscope. F4/80^+^ macrophage area within the aortic sinus was defined using the thresholding function of ImageJ (NIH, Bethesda, MD, USA).

### 2.7 Circulating and bone marrow leucocyte flow cytometry

Heparinized whole blood underwent erythrocyte lysis (Pharmalyse; BD Biosciences, Wokingham, UK) prior to isolation of leucocytes by centrifugation. Bone marrow was flushed from one femur and tibia using PBS with 0.5% BSA and 2 mM EDTA. After washing and resuspending in PBS with 0.5% BSA and 0.5% foetal calf serum (FCS), cells were incubated at 4°C with CD16/32 Fc block (BD Biosciences), then 10 min later with anti-CD45-VioBlue (Miltenyi Biotec, Woking, UK), anti-CD11b-FITC (Miltenyi Biotec), anti-Ly6G-PE (Miltenyi Biotec), and Ly6C-APC (eBioscience), for a further 10 min, prior to washing unbound antibodies. Some bone marrow samples were separately stained with anti-lineage cocktail-eFluor450 (eBioscience), anti-c-Kit-PE (Miltenyi Biotec), and anti-Sca-1-APC (Miltenyi Biotec) for 10 min. Flow cytometry (Fortessa, BD Biosciences) was performed to acquire leucocytes based on typical light scatter properties, with further gating used to define the following subsets: total leucocytes—CD45^+^; myeloid cells—CD45^+^CD11b^+^; monocytes—CD45^+^CD11b^+^Ly6G^−^Ly6C^+^; neutrophils—CD11b^+^Ly6G^hi−^Ly6C^hi^; ‘inflammatory’ monocytes—CD11b^+^Ly6G^−^Ly6C^hi^; ‘patrolling’ monocytes—CD11b^+^Ly6G^−^Ly6C^lo^; haematopoietic stem cells—Lin^−^Sca-1^+^c-Kit^+^. All populations are expressed as cells/mL for blood or cells/femur + tibia for bone marrow. Representative images of the gating strategies are provided in Supplementary material online, *Figure S12*.

### 2.8 Pulmonary endothelial cell isolation and culture

Primary endothelial cells were isolated from lungs by immunoselection with CD146-microbeads (Miltenyi Biotec), as previously reported.^28^ Bead-bound cells were magnetically separated from non-bead-bound cells using MS columns (Miltenyi Biotec) and resuspended in Endothelial growth medium–MV2 (PromoCell, Heidelberg, Germany) supplemented with hEGF (human Epidermal Growth Factor), hydrocortisone, vascular endothelial growth factor, hFGF-B, R3-IGF-1, ascorbic acid, gentamicin, amphotericin-B, and 5% FCS and seeded on fibronectin-coated plates. Cells were cultured at 37°C in 5% CO_2_ with twice-weekly media changes until confluent.

### 2.9 Immunoblotting

Primary pulmonary endothelial cells were lysed in extraction buffer containing 50 mM HEPES, 120 mM NaCl, 1 mM MgCl_2_, 1 mM CaCl_2_, 10 mM NaP_2_O_7_, 20 mM NaF, 1 mM EDTA, 10% glycerol, 1% NP40, 2 mM sodium orthovanadate, 0.5 µg/mL leupeptin, 0.2 mM PMSF, and 0.5 µg/mL aprotinin. Cell extracts were sonicated in an ice-bath and centrifuged for 15 min at 12 100 g, before protein measurements were carried out by BCA assay (Pierce Protein Quantification Kit, Thermo, Altrincham, UK) using the supernatant. Equal amounts of cellular protein were resolved on sodium dodecyl sulphate polyacrylamide gels (Invitrogen, Altincham, UK) and transferred to polyvinylidine difluoride membranes. Immunoblotting was carried out with indicated primary antibodies, diluted as necessary in 5% BSA-TBST buffer. Blots were incubated with appropriate horseradish peroxidase–conjugated secondary antibodies and developed with enhanced chemiluminescence (Merck). Fifty micrograms of total cell lysate were used for immunoprecipitation and 30 μg for western blotting with indicated antibodies: β-actin (Santa Cruz Biotechnology, Dallas, TX, sc-47778), HSP90 α/β (Santa Cruz Biotechnology, sc-13119), Nox2 (BD Biosciences, 611 414), Nox4 (Gift from Professor Ajay Shah^26^), IGF-1R (Cell Signaling Technology, 8521), phospho-IGF-1R (Cell Signaling Technology, 3918S), eNOS (Cell Signaling Technology, 9572), phospho-eNOS (Cell Signaling Technology, 9570), VCAM-1 (Abcam, ab174279), ICAM-1 (Abcam, ab25375), VE-Cadherin (Santa Cruz Biotechnology, sc-9989) phospho-Y731 VE-Cadherin (Sigma-Aldrich, Gillingham, UK, SAB4301448), Occludin (Thermo, 71-1500), Claudin-5 (Thermo, 34-1600), CD31 (DAKO, Stockport, UK, M0823), Akt (BD Biosciences, 610860), phospho-Akt (Cell Signaling Technology, 4060), p38 MAPK (Cell Signaling Technology, 9212), phospho-p38 MAPK (Cell Signaling Technology, 9216), JNK (Cell Signaling Technology, 9252), phospho-JNK (Cell Signaling Technology, 9255), Erk1/2 (Cell Signaling Technology, 4696), phospho-Erk1/2 (Cell Signaling Technology, 4370), YAP/TAZ (Cell Signaling Technology, 8418S), Histone H3 (Cell Signaling Technology, 4499T), and alpha-tubulin (Santa Cruz Biotechnology, c-5286). Immunoblots were scanned on a Syngene G:Box Chemi XT4 and quantified using the FIJI software package.

### 2.10 Real-time quantitative PCR

RNA was isolated using TRI-Reagent (Sigma) and cDNA prepared using a High-Capacity cDNA Reverse Transcription kit (Applied Biosystems, Altrincham, UK) according to the manufacturer’s instructions, as previously described.^28^ Real-time quantitative reverse transcription PCR was undertaken using an ABI-7500 System with specific Taqman primer/probe sets (Applied Biosystems) for detecting *Actb* (Mm00607939_s1), *IGF1R* (Hs00609566_m1), *Vcam1 (Mm01320970_m1)*, and *Icam1(Mm00516023_m1).* Data are expressed relative to *Actb* mRNA expression using the 2^−ΔCT^ method.

### 2.11 Bone marrow transplant studies

Sca-1^+^ bone marrow cells were isolated from the femurs and tibiae of 8-week-old ApoE^−/−^ or hIGFREO/ApoE^−/−^ donor mice using anti-Sca-1 microbeads, according to the manufacturer’s protocol (Miltenyi Biotec). Eight-week-old male ApoE^−/−^ mice were irradiated with a single dose of 8.45 Gy 1 day prior to receiving an intravenous injection of 1 × 10^6^ donor Sca-1^+^ bone marrow cells. Drinking water was supplemented with Enrofloxacin (0.005%) for 4 weeks after bone marrow transplantation as prophylaxis against infection. Recipient mice were then fed western diet for 12 weeks before assessing circulating leucocyte counts and aortic atherosclerosis using the methods described above. Confirmation of appropriate bone marrow engraftment was confirmed by assessing bone marrow leucocyte and Lin^−^Sca-1 ^+^ c-Kit^+^ (LSK) abundance, and by defining hIGFREO transgene in genomic DNA, as appropriate.

### 2.12 Bone marrow and femoral artery VE-Cadherin immunofluorescence

Mice received an intravenous injection of 12.5 μg Alexa Fluor® 647 conjugated anti-mouse VE-Cadherin antibody (Biolegend) and were euthanized 10 min later. Femurs were immediately fixed in 4% paraformaldehyde in PBS and placed on ice for 4 h, decalcified in 0.5 M EDTA at 4°C for 24 h, and then cryoprotected in a solution of 20% sucrose and 2% polyvinylpyrrolidone at 4°C for 24 h.^33^ Bones were then placed in embedding solution [8 g gelatin (Sigma-Aldrich), 2 g PVP, and 20 g sucrose in 80 mL PBS], and then stored at −80°C, before cryosectioning (Leica CM3050S) at 70 μm thickness. Sections were mounted on slides with DAPI Fluoromount-G® counterstain (Southern Biotech, Birmingham, AL). Femoral arteries were cut longitudinally and mounted *en face* on a slide with DAPI Fluoromount-G® counterstain. All samples were imaged on a Zeiss LSM880 confocal microscope. ImageJ (NIH) was used to define a thresholded VE-Cadherin stained area percentage in bones and femoral arteries. Thresholded images of the femoral artery were further utilized to create a distance map, the data from which were summarized using the histogram function, which describes the number of pixels required to move from VE-Cadherin positive to negative for every VE-Cadherin positive pixel; this was used to infer a junctional thickness profile.

### 2.13 Aortic endothelial BODIPY-LDL uptake

Mice received an intravenous injection of 50 μg BODIPY-LDL (Thermo; L3483) and were euthanized by terminal exsanguination whilst under inhaled 4% isoflurane anesthesia 30 min later. After perfusion fixation with 4% paraformaldehyde in PBS, the aorta was dissected free, cut longitudinally, and mounted *en face* on a slide with DAPI Fluoromount-G® counterstain. All samples were imaged on a Zeiss LSM880 confocal microscope. ImageJ (NIH) was used to define a thresholded BODIPY stained area percentage.

### 2.14 Evans blue vascular permeability assay

Mice were anaesthetized with inhaled 4% isoflurane, and 100 µL 5% Evans blue (Sigma-Aldrich) was injected intravenously into the tail vein. After 15 min the mouse was euthanized by terminal exsanguination, and perfused with 25 mL PBS, via cardiac puncture to clear all vascular-resident dye. The whole aorta (root to abdominal bifurcation), brain, femur, tibia, and gastrocnemius/soleus muscle were harvested. These were dried overnight at 55°C, then weighed and placed into 1 mL formamide for 24 h at 55°C. The femur and tibia were cut into small pieces before placing into the formamide (Sigma-Aldrich). After 24 h, samples were centrifuged at 1000*g* for 5 min. A concentration curve was created for Evans Blue. These were transferred to a 96-well plate with duplicate 100 µL samples. Absorbance of all samples was measured at 620 nm using a Thermo Multiskan™ GO Microplate Spectrophotometer (Thermo, Altrincham, UK) and adjusted using formamide absorbance as a blank. The concentration of Evans Blue was calculated using the concentration curve and then standardized to dry sample weight. Individual mouse data were normalized within experimental batches to the wild-type (WT) batch mean.

### 2.15 Generation of lentivirus particles, titre quantification, and cell transduction

Human IGF-1R cDNA was sub-cloned into the vector pLVX-TetOne™ (TaKaRa, San Jose, CA) allowing for doxycycline-regulated protein expression. The sequence of human WT IGF-1R and its K1003R mutant were verified by sequencing (DNA Sequencing & Services, MRC PPU, University of Dundee). For the generation of lentivirus particles, HEK-293 LentiX™ cells were seeded at 5 × 10^6^ per 100 mm dish in 10 mL of Dulbecco’s Modified Eagle’s Medium/Glutamax (Thermo) supplemented with 10% tetracycline-free FCS (TaKaRa), 100 U/mL penicillin, 100 μg/mL streptomycin, 0.25 μg/mL amphotericin B (Merck). The next day cells were transfected using Lenti-X™ Packaging Single Shots (VSV-G; TaKaRa) following the manufacturer’s instructions. The obtained lentivirus supernatant was concentrated using Lenti-X™ Concentrator solution (TaKaRa) following the manufacturer’s instructions. The lentivirus supernatant was stored at −80°C. For quantification of the lentivirus titre, a lentivirus-associated p24 ELISA kit (Cell Biolabs, San Diego, CA) was used. Human umbilical vein endothelial cells (HUVECs) were transduced at a multiplicity of infection (MOI) of 10 for 16–18 h before induction of protein expression with doxycycline hyclate (Merck) for 48–72 h.

### 2.16 Human umbilical vein endothelial cell studies

Passage 3–4 HUVEC (PromoCell) from at least three different donors were cultured in Endothelial Cell Growth Medium 2 (PromoCell) supplemented with 1× Antibiotic Antimycotic Solution (Merck) at 37°C in 5% CO_2_. For transduction with lentivirus particles coding for WT or kinase-dead (K1003R) human IGF-1R under a doxycycline-inducible promoter using a MOI of 10, 16–18 h later, IGF-1R expression was induced with doxycycline (2 µg/mL) for 72 h. *Immunoblotting studies:* Cells were lysed in Cell Extraction Buffer (Invitrogen) supplemented with Protease Inhibitor Cocktail (Merck). Fifteen micrograms cell lysate were loaded on a 4–12% NuPAGE Bis-Tris gel (Thermo), resolved by electrophoresis, and subsequently transferred on to a Nitrocellulose membrane using the TransBlot Turbo™ system (BioRad, Watford, UK). For preparation of nuclear and cytosolic lysates used for immunoblotting, see details in nuclear factor kappa B (NF-κB) activity assay. Ten micrograms lysate were used for cytoplasmic and nuclear immunoblotting*. Immunocytochemistry:* Confluent HUVECs were fixed with pre-warmed 2% paraformaldehyde for 5 min (or with methanol for 10 min at −20°C for claudin-5 experiments), permeabilized with 0.1% TritonX-100 for 5 min at room temperature, and non-specific sites were blocked using 5% donkey serum/PBS for 60 min at room temperature. Primary antibody incubation (VE-Cadherin, clone BV-9, BioLegend 348502, 5 µg/mL; Claudin-5 Invitrogen 34-1600, 1 µg/mL; Caveolin-1, Cell Signaling Technology 3267, 1:200 dilution; clathrin heavy chain, Cell Signaling Technology 4796, 1:100 dilution; ApoB, Thermo MIA1605, 2 µg/mL) was in 1% donkey serum/PBS overnight at 4°C. Secondary antibody incubation was in 1% donkey serum/PBS for 60 min at room temperature. Cells were mounted with ProLong™ Diamond with DAPI (Thermo). Cells were visualized using an LSM880 microscope (Zeiss, Cambridge, UK). *Permeability studies:* HUVECs were seeded to confluence at 1 × 10^5^/cm^2^ into 6.5 mm diameter PET-inserts with 0.4 µm pores (Corning). After 72 h, the confluent monolayers were washed in ECGM2, and 1 h later were stimulated on the apical side with PBS or 100 µM H_2_O_2_ for 20 min in ECGM2 with 2% FCS prior to apical addition of 50 µg 40 kDa FITC-dextran in ECGM2 with 2% FCS with or without 100 µM H_2_O_2_ for a further 20 min. Medium was collected from the basal side and fluorescence recorded using 480 nm excitation and 520 nm emission using a Synergy H1 plate reader (Biotek, Santa Clara, CA). *LDL-cholesterol uptake studies:* Unlabelled or BODIPY-labelled LDL-cholesterol (Thermo) was applied to HUVEC at a concentration of 10 µg/mL in ECGM2 for 30 min prior to washing twice with PBS and fixation with pre-warmed 2% paraformaldehyde for 5 min. Unlabelled LDL was visualized with ApoB immunocytochemistry, as described above. BODIPY-labelled LDL was visualized directly using an LSM880 microscope (Zeiss). *NF-κB activity assay:* HUVECs were grown, transduced with WT or K1003R IGF-1R lentivirus particles, and expression induced as described above. After 72 h, cells were trypsinized, collected by centrifugation at 4°C and stored at −80°C in freezing medium Cryo-SFM (PromoCell). Untransduced HUVEC treated with PBS (Merck) or 10 ng/mL tumour necrosis factor alpha (TNF-α; Biotechne) for 4 h followed by storage in Cryo-SFM at −80°C served as control for NF-κB activation. To obtain the nuclear extract, Nuclear Extraction Kit (Abcam, ab113474) was used following the manufacturer’s protocol. To measure NF-κB activity, NF-κB p65 Transcription Factor Assay Kit (Abcam, ab133112) was used following the manufacturer’s protocol and using 5 μg nuclear extract per condition. *Analysis of cell surface ICAM1 and VCAM1 expression by flow cytometry:* HUVECs were grown, transduced with WT or K1003R IGF-1R lentivirus particles, and expression induced as described above. After 72 h, cells were detached with 0.48 mM EDTA in PBS and gentle scraping, collected by centrifugation at 4°C and immuno-labelled with APC-conjugated anti-ICAM1 (Miltenyi Biotec, 130-121-427) and FITC-conjugated anti-VCAM1 (Miltenyi Biotec, 130-124-703); positive control HUVECs were exposed to 10 ng/mL TNF-α (Biotechne) for 4 h instead of lentiviral transduction. Analysis was performed using a Beckman Coulter CytoFLEX flow cytometer, collecting data from at least 10 000 singlet cells. Representative images of the gating strategies are provided in Supplementary material online, *Figure S12*. *RNA-seq:* RNA was isolated from confluent HUVEC over-expressing WT or K1003R IGF-1R using TRIzol (Thermo) and the RNA Clean & Concentrator-25 kit (Zymo Research, Cambridge, UK) with DNAse I treatment. RNA-seq was performed by the University of Leeds Next Generation Sequencing core facility (Illumina NextSeq 2000) to acquire 100 bp single-end reads. Raw data have been deposited at ArrayExpress (https://www.ebi.ac.uk/biostudies/arrayexpress) under accession ID E-MTAB-14458.

### 2.17 Bioinformatics

Quality control of the raw sequences was performed using FastQC v.0.11.4 to evaluate the overall quality.^34^ Adapter sequences were trimmed from raw reads (TrimGalore v.0.6.6),^35^ before alignment to the mouse genome (GRCm39) using STAR aligner v.2.7.10a followed by featureCounts (Subread v2.0.1) to derive gene count data.^36^ Subsequent bioinformatics were conducted using DESeq2 v.1.36.0 in the R environment (v4.2.0) for differential gene expression analysis,^37^ and g:Profiler (https://biit.cs.ut.ee/gprofiler/gost) for defining enriched Gene Ontology (GO) terms amongst differentially expressed genes (DEGs). Multiple testing was accounted for using Benjamini–Hochberg false discovery rate (FDR) adjusted *P*-values, both when defining DEGs and enriched GO terms.

### 2.18 Statistics

Data were analysed using GraphPad Prism 10 (GraphPad Software Inc., Boston, MA). The results are expressed as mean [standard error of the mean (SEM)]. Comparisons within groups are made using paired Student’s *t*-tests, and between groups using unpaired Student’s *t*-tests or repeated measures analysis of variance as appropriate. Where repeated *t*-tests are performed, a Bonferroni correction is applied. *P* < 0.05 is considered statistically significant, and all statistical tests are two sided. *n* denotes the number of mice per experiment, or the number of animals where cells are used, or the number of human donors (i.e. independent experimental replicates). Researchers were blinded to genotype or treatment allocation in all experiments.

## 3. Results

### 3.1 hIGFREO/ApoE^−/−^ mice develop less atherosclerotic plaque

To generate atherosclerosis-prone mice with increased endothelial expression of IGF-1R, we crossed previously described ‘hIGFREO’ mice (with endothelium-specific transgenic expression of human IGF-1R)^25^ with ApoE knockout mice to generate hIGFREO/ApoE^−/−^. hIGFREO/ApoE^−/−^ and ApoE^−/−^ littermates were born at frequencies compatible with Mendelian inheritance and appeared morphologically normal. There was no difference in body weight between hIGFREO/ApoE^−/−^ and ApoE^−/−^ littermates prior to western diet feeding (at 8 weeks of age) or at study completion (at 20 weeks of age; see Supplementary material online, *Figure S1A*). Total IGF-1R expression was significantly increased in lung endothelial cells from hIGFREO/ApoE^−/−^ (see Supplementary material online, *Figure S1B*), and human IGF-1R mRNA was not expressed in non-endothelial cells of hIGFREO/ApoE^−/−^ lungs (see Supplementary material online, *Figure S1C*). Human IGF-1R mRNA was barely detectable (>100-fold lower than fresh aorta) in circulating CD11b^+^ cells from hIGFREO/ApoE^−/−^ (see Supplementary material online, *Figure S1D*), demonstrating minimal Tie2 promoter-induced transgene expression in the myeloid compartment.

**Figure 1:**
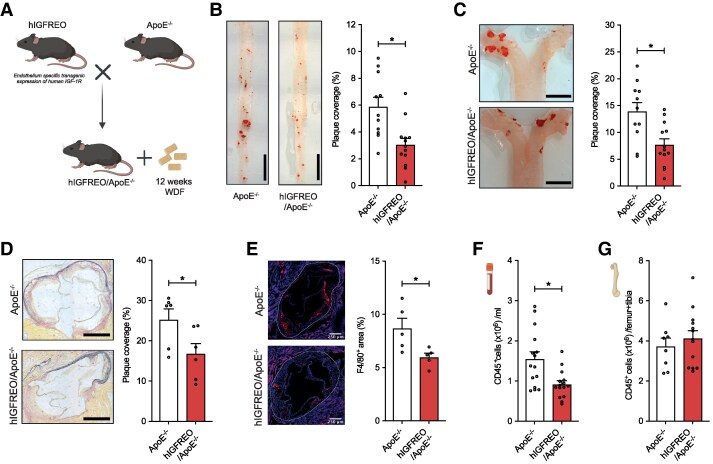
hIGFREO/ApoE^−/−^ mice develop less atherosclerotic plaque. (*A*) Schema of hIGFREO/ApoE^−/−^ generation. In comparison with ApoE^−/−^ littermates, hIGFREO/ApoE^−/−^ develop: (*B*) reduced atherosclerotic plaque area defined by Oil-Red O staining in thoraco-abdominal aorta (*n* = 11, 13); scale bar denotes 5 mm; (*C*) reduced atherosclerotic plaque area defined by Oil-Red O staining in aortic arch (*n* = 11, 13); scale bar denotes 2000 μm; (*D*) reduced atherosclerotic plaque area in the aortic sinus (*n* = 6, 6); scale bar denotes 500 μm; (*E*) F4/80^+^ macrophage abundance (*n* = 5, 5); scale bar denotes 250 μm; (*F*) circulating CD45^+^ leucocytes (*n* = 15, 15); (*G*) bone marrow total CD45^+^ leucocytes (*n* = 8, 13). Data expressed as mean (SEM). *P < 0.05. *n* denotes the number of mice per group. All statistical comparisons are made with unpaired Student’s *t*-tests.

After 12 weeks western diet feeding (*Figure 1A*), hIGFREO/ApoE^−/−^ developed significantly less atherosclerosis in the thoraco-abdominal aorta (*Figure 1B*), aortic arch (*Figure 1C*), and aortic sinus (*Figure 1D*), compared with ApoE^−/−^ littermates. Immunofluorescence analysis of the aortic sinus also revealed reduced F4/80^+^ macrophage content in hIGFREO/ApoE^−/−^ (*Figure 1E*). However, expression of the leucocyte adhesion molecules VCAM-1 and ICAM-1 was similar in aortae (see Supplementary material online, *Figure S2A*–*D*) and isolated pulmonary EC (see Supplementary material online, *Figure S2E* and *F*) from hIGFREO/ApoE^−/−^ and control littermates.

Since IGF-1 is an established regulator of systemic metabolism, we performed a detailed characterization of systemic glucose metabolism and serum lipid profiles. After 12 weeks of western diet feeding, there was no difference in fasting glucose (see Supplementary material online, *Figure S3A*), glucose tolerance (see Supplementary material online, *Figure S3B*), insulin tolerance (see Supplementary material online, *Figure S3C*), fasting serum insulin concentration (see Supplementary material online, *Figure S3D*), or fasting IGF-1 concentration (see Supplementary material online, *Figure S3E*). Furthermore, there was no difference in fasting serum triglyceride (see Supplementary material online, *Figure S3F*) or cholesterol profile (see Supplementary material online, *Figure S3G*) between hIGFREO/ApoE^−/−^ and ApoE^−/−^ littermates. Hence, altered systemic metabolism did not appear to explain altered atherosclerosis in hIGFREO/ApoE^−/−^.

As IGF-1 receptors influence endothelial NO and ROS production, we next asked whether changes in these atherosclerosis-modulating factors were apparent in hIGFREO/ApoE^−/−^. After 12 weeks western diet, there was no difference in the systolic blood pressure of hIGFREO/ApoE^−/−^ vs. control littermates (see Supplementary material online, *Figure S4A*). *Ex vivo* assessment of aortic vasomotion found no significant differences in endothelium-dependent and endothelium-independent vasorelaxation in response to acetylcholine (see Supplementary material online, *Figure S4B*) or SNP (see Supplementary material online, *Figure S4C*), respectively. Moreover, we found similar constrictor responses to phenylephrine (see Supplementary material online, *Figure S4D*) and the non-selective NOS inhibitor L-NMMA (see Supplementary material online, *Figure S4E*), indicative of comparable NO biogenesis in response to isometric tension (quantified in Supplementary material online, *Figure S4F*). To explore the possibility that generation of ROS influenced vascular function and atherogenesis, we quantified acetylcholine-mediated vasodilation in the presence of the superoxide dismutase mimetic MnTMPyP and found no differences (see Supplementary material online, *Figure S5A*). Expression of NADPH oxidases NOX2 and NOX4, both important sources or ROS in EC, were also similar in hIGFREO/ApoE^−/−^ and ApoE^−/−^ aortae (see Supplementary material online, *Figure S5B*) and pulmonary EC (see Supplementary material online, *Figure S5C*). Since IGF-1R signals downstream to the NO generating enzyme eNOS, we also quantified basal expression of eNOS, and the ratio of ‘activated’ S1177-phosphorylated eNOS to total eNOS and elicited no differences (see Supplementary material online, *Figure S5D*). Moreover, we found no difference in insulin- or IGF-1-stimulated aortic eNOS activity in hIGFREO/ApoE^−/−^ compared with ApoE^−/−^ EC (see Supplementary material online, *Figure S5E*,*F*). Therefore, altered NO or ROS abundance appeared unlikely to explain the reduced atherosclerosis of hIGFREO/ApoE^−/−^ mice.

Next, we quantified circulating leucocytes as key mediators of atherosclerosis. Compared with ApoE^−/−^ littermates, hIGFREO/ApoE^−/−^ had a statistically significant 41% reduction in total circulating leucocytes (*Figure 1F*), which was consistent in magnitude across subsets including: CD45^+^CD11b^+^ myeloid cells (see Supplementary material online, *Figure S6A*), CD45^+^CD11b^+^Ly6G^−^Ly6C^+^ total monocytes (see Supplementary material online, *Figure S6B*; *P* = 0.069), CD11b^+^Ly6G^−^Ly6C^hi^ ‘inflammatory’ monocytes (see Supplementary material online, *Figure S6C*), CD11b^+^Ly6G^−^Ly6C^lo^ ‘patrolling’ monocytes (see Supplementary material online, *Figure S6D*; *P* = 0.16), and CD11b^+^Ly6G^+^Ly6C^hi^ neutrophils (see Supplementary material online, *Figure S6E*). The ratio of ‘inflammatory’ to ‘patrolling’ monocytes was not significantly different (see Supplementary material online, *Figure S6F*). Compared with ApoE^−/−^ littermates, hIGFREO/ApoE^−/−^ had similar bone marrow total leucocytes (*Figure 1G*), myeloid cells (see Supplementary material online, *Figure S6G*), monocytes (see Supplementary material online, *Figure S6H*), and neutrophils (see Supplementary material online, *Figure S6I*). Furthermore, we observed no difference in the abundance of LSK haematopoietic stem cells (see Supplementary material online, *Figure S6J*). These data imply that leucocytes are being appropriately generated in the bone marrow, but are not entering the systemic circulation.

To corroborate that undetected alterations in haematopoietic cells of hIGFREO/ApoE^−/−^ did not explain their reduced circulating leucocytes and atherosclerosis, we conducted bone marrow transplantation studies. Donor bone marrow Sca-1^+^ cells from hIGFREO/ApoE^−/−^ or ApoE^−/−^ control mice were transplanted into irradiated ApoE^−/−^ recipients, which were then fed western diet for 12 weeks after a 4-week period of recovery (see Supplementary material online, *Figure S7A*). Appropriate engraftment of hIGFREO/ApoE^−/−^ donor bone marrow was confirmed at the end of the experiment by detecting hIGF-1R genomic DNA in bone marrow. We observed no differences in total leucocyte count (see Supplementary material online, *Figure S7B*), CD45^+^CD11b^+^ myeloid cells as a proportion of total leucocytes (see Supplementary material online, *Figure S7C*), or ‘inflammatory’ to ‘patrolling’ monocyte ratio (see Supplementary material online, *Figure S7D*). Moreover, there was no difference in bone marrow resident total leucocytes (see Supplementary material online, *Figure S7E*), CD45^+^CD11b^+^ myeloid cells as a proportion of total leucocytes (see Supplementary material online, *Figure S7F*), or LSK haematopoietic stem cells as a proportion of bone marrow cells (see Supplementary material online, *Figure S7G*). Aortic atherosclerotic plaque area was also unaltered in ApoE^−/−^ recipients of hIGFREO/ApoE^−/−^ bone marrow (see Supplementary material online, *Figure S7H*). Therefore, off-target effects in haematopoietic cells do not account for the phenotype of hIGFREO/ApoE^−/−^ mice.

### 3.2 hIGFREO/ApoE^−/−^ mice have lower endothelial paracellular and transcellular permeability

**Figure 2:**
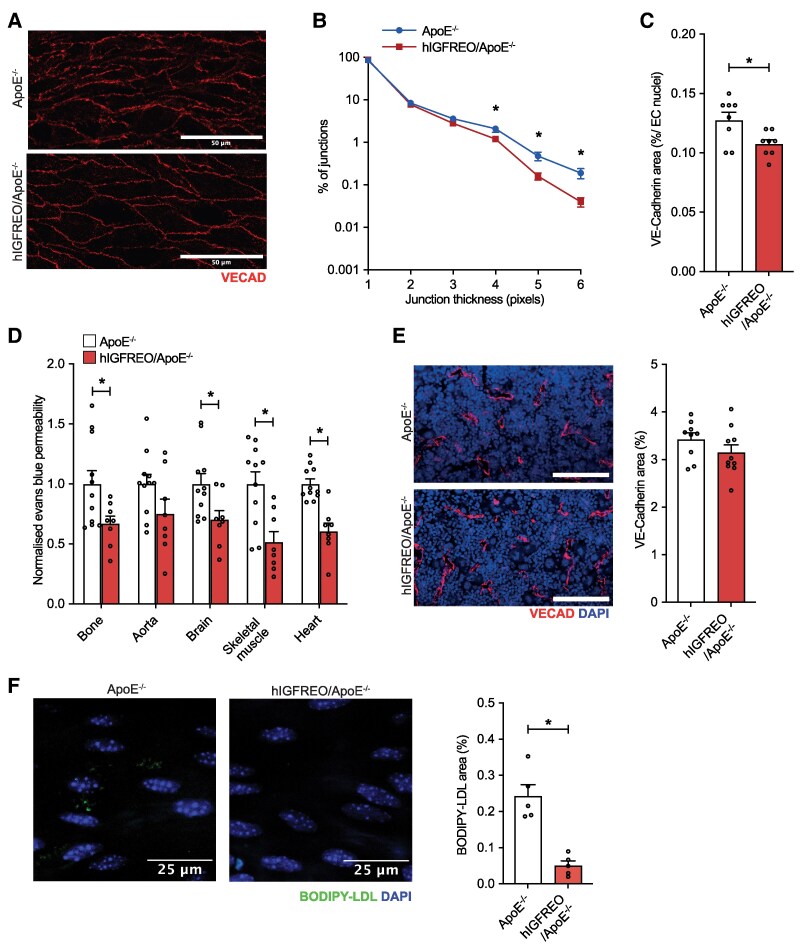
hIGFREO/ApoE^−/−^ exhibit morphological features of VE-Cadherin junction stabilization, reduced vascular paracellular permeability, and reduced endothelial LDL-cholesterol uptake. (*A*) Representative images of femoral artery VE-Cadherin junctions in hIGFREO/ApoE^−/−^ and ApoE^−/−^ controls (scale bar denotes 50 μm). (*B*) VE-Cadherin junctions are thinner in hIGFREO/ApoE^−/−^. (*C*) VE-Cadherin junction area is lower in hIGFREO/ApoE^−/−^ (*n* = 8, 8). (*D*) Vascular permeability of Evans Blue dye is reduced in multiple vascular beds of hIGFREO/ApoE^−/−^ (*n* = 11, 8). (*E*) Vascularity of bone marrow is similar in hIGFREO/ApoE^−/−^ and ApoE^−/−^ controls; in representative images, red denotes VE-Cadherin (VECAD) and blue denotes DAPI (*n* = 9, 10); scale bar denotes 100 μm. (*F*) Uptake of BODIPY-labelled LDL-cholesterol is reduced in the aortic endothelium of hIGFREO/ApoE^−/−^ at 8 weeks of age without western diet feeding (representative images on left show BODIPY in green and DAPI in blue; *n* = 5, 5); scale bar denotes 25 μm. Data expressed as mean (SEM). *P < 0.05. *n* denotes the number of mice per group; all statistical comparisons are made with unpaired Student’s *t*-tests.

We next focused on endothelial junctions, given their proposed dysfunction in early atherogenesis, leading to subendothelial transit of LDL-cholesterol and leucocytes.^24^ Perfusion of mice with a fluorophore tagged anti-VE-Cadherin antibody revealed that arterial VE-Cadherin junctions appeared thinner and more organized in hIGFREO/ApoE^−/−^ vs. ApoE^−/−^ (*Figure 2A*). Quantification of this confirmed that thicker VE-Cadherin junctions were significantly more common in ApoE^−/−^ controls (*Figure 2B*), in association with a larger VE-Cadherin stained surface area (*Figure 2C*); this phenotype is known to be linked with reduced vascular leakage.^38^ To define vascular permeability in multiple vascular beds, including the bone marrow and aorta, we perfused mice with Evans Blue dye and quantified extravascular leakage; this revealed significantly reduced leakage in multiple vascular beds of hIGFREO/ApoE^−/−^ (*Figure 2D*). Importantly, this was not associated with any reduction in bone marrow vascularity (*Figure 2E*), suggesting the reduced bone marrow vascular permeability of hIGFREO/ApoE^−/−^ relates to altered vessel function, not abundance. Hence, increased endothelial IGF-1R over-expression induces a leakage resistant endothelial junction morphology and reduces vascular permeability in multiple vascular beds.

Whilst endothelial junctions are an important regulator of LDL-cholesterol transit into the arterial wall, transcellular transport across EC also makes a substantial contribution.^24,39^ Indeed, in contrast with arterial endothelium, brain endothelium transports very little circulating LDL-cholesterol to its abluminal surface;^23,40^ IGF-1R expression is also enriched in brain EC.^8–10^ Moreover, recent data have linked IGF-1R expression in brain EC to the extent of their selective transcytosis of circulating molecules.^41^ Hence, to complement our Evans Blue dye leakage studies, we also studied vascular uptake of infused atherogenic lipoproteins. Eight-week-old hIGFREO/ApoE^−/−^ mice fed a normal chow diet received intravenous injection of fluorescent BODIPY-LDL-cholesterol prior to visualizing its accumulation in the aortic endothelial monolayer 30 min later. This revealed markedly lower LDL fluorescence in the aortic endothelium of hIGFREO/ApoE^−/−^ vs. control littermates (*Figure 2F*), indicating that lower transcellular permeability to LDL may contribute to their lower atherosclerotic plaque area after feeding with a high cholesterol diet.

### 3.3 IGF-1R kinase activity is required for its permeability modulating effects

**Figure 3:**
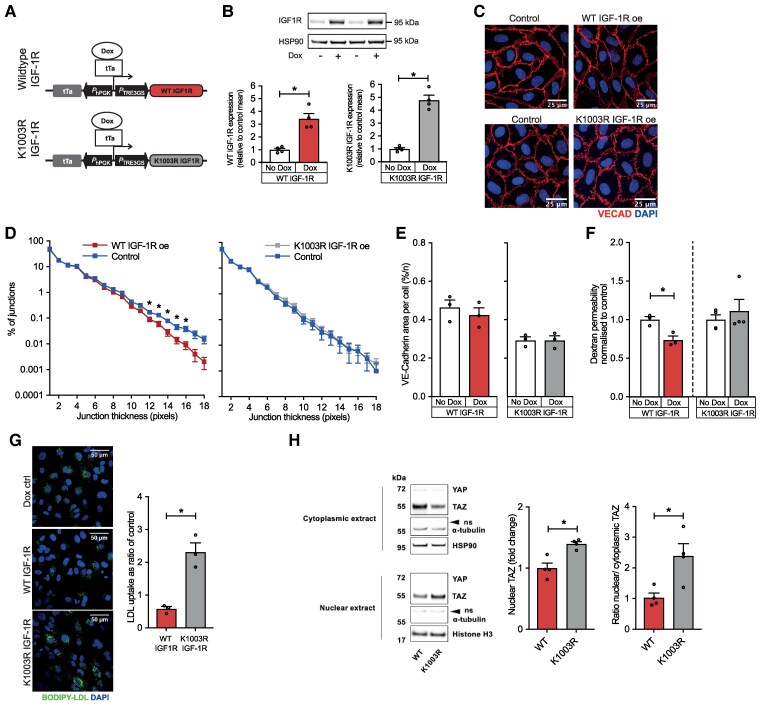
IGF-1 receptor over-expression reduces paracellular leakage and LDL-cholesterol uptake in human endothelial cells via its kinase domain. (*A*) Schema of lentiviral constructs used to achieve doxycycline-inducible WT or kinase-dead (K1003R) IGF-1R over-expression. (*B*) WT and K1003R IGF-1R protein expression are increased approximately three-fold by doxycycline. (*C*) Representative images of HUVEC VE-Cadherin with either WT or K1003R IGF-1R over-expression (VE-Cadherin—red, DAPI—blue). *D*) VE-Cadherin junction thickness is reduced in confluent HUVEC exposed to 100 μM hydrogen peroxide over-expressing WT IGF-1R, but remains unchanged with cells over-expressing K1003R IGF-1R. (*E*) VE-Cadherin junction area is similar in confluent HUVEC over-expressing WT or K1003R IGF-1R. (*F*) Paracellular leakage of 40 kDa FITC-Dextran is reduced in HUVEC over-expressing WT IGF-1R, but not K1003R, IGF-1R. *G*) Uptake of BODIPY-labelled LDL-cholesterol is reduced in HUVEC over-expressing WT IGF-1R, but not K1003R, IGF-1R (representative images on left; BODIPY—green, DAPI—blue). (*H*) Representative immunoblots of YAP/TAZ and loading controls in nuclear and cytosolic lysates, accompanied by quantification of normalized nuclear TAZ expression and nuclear:cytosolic TAZ ratio, showing reduced nuclear TAZ in HUVEC over-expressing WT vs. K1003R IGF-1R. ns denotes non-specific band in α-tubulin blot. Data expressed as mean (SEM). *P < 0.05; *n* = 3, 3 in a–g and *n =* 4, 4 in h. All statistical comparisons are made with unpaired Student’s *t*-tests.

To define the relevance of these findings to human vascular biology and to explore underpinning mechanisms, we over-expressed IGF-1R in human endothelium. HUVECs were transduced with lentivirus particles, allowing doxycycline-inducible WT IGF-1R over-expression (*Figure 3A*); transduced cells not exposed to doxycycline served as control. To define whether the signalling function of the IGF-1R transgene is essential for its functional effects, we also repeated experiments using an identical lentivirus system allowing doxycycline-inducible K1003R IGF-1R expression; this mutation in the ATP-binding domain renders IGF-1R kinase inactive.^26^ This system achieved a near three-fold increase in IGF-1R protein (*Figure 3B*), broadly mimicking our murine model (see Supplementary material online, *Figure S1B*). Cells were grown to confluence and exposed to 100 μM hydrogen peroxide to model the permeability-inducing conditions observed in atherosclerosis. In comparison with control cells, WT IGF-1R over-expression led to thinner VE-Cadherin junctions and reduced VE-Cadherin junction area; no such effect was observed with K1003R IGF-1R over-expression (*Figure 3C–E*). Importantly, leakage of FITC-Dextran across a confluent monolayer of WT IGF-1R over-expressing HUVEC was substantially reduced vs. control cells, whereas K1003R IGF-1R over-expression had no effect (*Figure 3F*). Hence, the kinase activity of IGF-1R is required for its influence on paracellular leakage between endothelial junctions, implying that its downstream signalling cascade is required.

Liang *et al.* have shown that mice with endothelial deletion of IGF-1R exhibit increased renal leucocyte infiltration after ureteric obstruction, associated with increased phosphorylation of the endothelial cell–cell junction protein VE-Cadherin at Y731.^21^ Moreover, Higashi *et al*. have found that deletion of endothelial IGF-1R increases atherosclerosis in mice, associated with a trend towards increased vascular leakage *in vivo* and decreased expression of multiple EC junction proteins *in vitro*.^22^ Therefore, we immunoblotted for EC junction proteins using our *in vitro* HUVEC model. Over-expression of WT IGF-1R was not associated with increased expression of the junction proteins Claudin-5, CD31, Occludin or VE-Cadherin; similarly, over-expression of K1003R IGF-1R did not alter expression of these proteins (see Supplementary material online, *Figure S8A*). Notably, we also found no difference in VE-Cadherin Y731 phosphorylation in IGF-1R over-expressing HUVEC (see Supplementary material online, *Figure S8B*). However, we noted increased Claudin-5 junctional area in WT, but not K1003R, IGF-1R over-expressing HUVEC using immunofluorescence (see Supplementary material online, *Figure S8C*). These data are consistent with IGF-1 signalling altering the localization, but not expression, of junctional proteins (VE-Cadherin and Claudin-5), perhaps by altering junctional protein turnover, resulting in stabilized cell–cell junctions.

To address the transcellular route, we again performed BODIPY-LDL uptake experiments in HUVEC over-expressing WT or K1003R IGF-1R. These revealed a reduction in LDL uptake in cells expressing WT IGF-1R vs. non-transduced cells, whilst the converse was observed in K1003R IGF-1R expressing cells (*Figure 3G*). To explore whether this reflected altered interaction of LDL-cholesterol with clathrin or caveolin coated vesicles—the two major routes of transcytosis—we defined co-localization between proteins coating these vesicles and the LDL-cholesterol intrinsic protein ApoB. We found only a small proportion of ApoB co-localized with caveolin-1 and this was not altered by WT or K1003R IGF-1R over-expression. However, we noted a significant increase in the co-localization of ApoB with clathrin heavy chain when WT IGF-1R was over-expressed, whilst K1003R over-expression did not alter this (see Supplementary material online, *Figure S9*). This may imply that altered behaviour of clathrin-mediated vesicle transport underpins the lower quantity of BODIPY-LDL internalized by WT IGF-1R over-expressing HUVEC.

To explore signalling events that may underpin the phenotype observed in HUVEC over-expressing WT IGF-1R, we performed immunoblotting of total and phosphorylated IGF-1R and kinases in its major downstream pathways. As shown in Supplementary material online, *Figure S10A* and *B*, there was a non-significant 2-fold increase in phosphorylated IGF-1R in WT IGF-1R over-expressing cells, which was not observed with K1003R IGF-1R expression. Neither WT nor K1003R over-expression altered the expression of total or phosphorylated Akt, nor ERK, JNK, and p38 MAP kinases. An NF-κB activity assay also revealed no change in activation induced by WT or K1003R IGF-1R (see Supplementary material online, *Figure S10C*), despite robust activation by TNF-α. Similarly, flow cytometry revealed no difference in cell surface localized adhesion molecules VCAM-1 and ICAM-1 (see Supplementary material online, *Figure S10D*).

This led us to perform an unbiased assessment of WT vs. K1003R IGF-1R over-expressing HUVEC using RNA-seq to guide our analyses. Comparison of data from three samples per group revealed no hits achieving FDR adjusted *P*–values <0.05, but did yield 48 hits with unadjusted *P* < 0.05 (*Table 1* and Supplementary material online, Data  *S1*). Functional enrichment analysis of the 48 hits (see Supplementary material online, Data  *S2*) revealed GO terms including ‘Positive regulation of signal transduction’ (GO:0009967) and ‘Actin filament-based process’ (GO:0030029), but did not highlight specific molecular pathways to pursue. However, we noted multiple genes regulated by YAP/TAZ transcription factors, a process not encapsulated by GO terms. Therefore, we defined over-expression of YAP/TAZ target genes using a published list of 22 targets,^42^ finding four of these within our 48 hits (χ^2^ statistic 374.9; *P* < 1 × 10^−8^). These four gene hits (ANKRD1, CTGF, CYR61, and AXL) were downregulated in WT vs. K1003R IGF-1R expressing HUVEC, leading us to hypothesize diminished nuclear YAP/TAZ localization in WT IGF-1R over-expressing cells. To test this, we immunoblotted YAP and TAZ in nuclear and cytosolic lysates from of WT vs. K1003R IGF-1R expressing HUVEC and confirmed a lower nuclear abundance and lower nuclear:cytosolic ratio of TAZ in WT over-expressing cells (*Figure 3H*). These data suggest that WT IGF-1R suppresses endothelial nuclear TAZ localization, a phenomenon previously associated with reduced atherogenesis.^43^

**Table 1:** Differentially expressed genes in WT IGF-1R vs. K1003R IGF-1R over-expressing HUVEC

Gene	Log_2_ fold change	Unadjusted *P*-value
ANKRD1	−0.24	0.0004
POTEE	1.34	0.0007
MSMO1	−0.24	0.0053
CFL2	−0.24	0.0061
ESM1	−0.19	0.0108
STC1	0.42	0.0129
MT-ATP8	−0.27	0.0138
RFNG	0.35	0.0173
AC008750.5	2.96	0.0174
LINC01145	−2.43	0.0196
RNU6-100P	−3.56	0.0197
AC004241.5	2.41	0.0198
AC037198.1	0.18	0.0240
COL12A1	−0.14	0.0267
AC040904.1	−3.77	0.0288
ANLN	−0.15	0.0292
CDH2	−0.17	0.0295
GADD45B	−0.31	0.0295
CTGF	−0.12	0.0297
NDUFAF4P3	−3.90	0.0298
HOXC8	−1.85	0.0299
CYR61	−0.12	0.0301
ANKRD6	0.37	0.0303
AC012313.1	0.55	0.0311
RN7SKP198	−3.25	0.0316
AC003080.1	0.47	0.0325
AXL	−0.15	0.0329
PLOD2	−0.14	0.0348
SERPINE1	−0.11	0.0373
ABTB1	0.43	0.0376
DPM1	−0.28	0.0381
AC084024.1	−1.55	0.0383
PRPF4	−0.24	0.0400
ALCAM	−0.13	0.0403
AJM1	−1.00	0.0405
CCAT2	2.34	0.0427
TFRC	−0.13	0.0442
ZAR1	2.53	0.0448
TGFB2	−0.27	0.0452
AC125257.1	−0.34	0.0457
TMSB15B	1.66	0.0462
MME-AS1	2.66	0.0462
LSM3	−0.28	0.0470
RPL26P27	1.49	0.0472
GBA2	0.15	0.0481
PSAT1	−0.20	0.0492
RPL31P52	1.83	0.0498
CALU	−0.13	0.0498

Differentially expressed genes achieving unadjusted statistical significance between human umbilical vein endothelial cells over-expressing WT vs. K1003R IGF-1R. Complete data from this RNA-seq analysis are presented in Supplementary material online, Data  *S1*.

**Figure 4:**
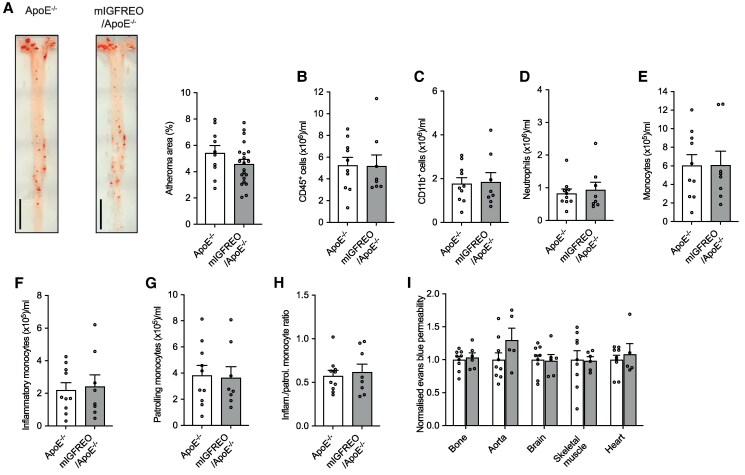
ApoE^−/−^ mice with endothelial-specific over-expression of the kinase-dead K1003R mutant IGF-1R (mIGFREO/ApoE) exhibit comparable atherosclerosis, circulating leucocytes and vascular permeability to ApoE^−/−^ mice. After 12 weeks of western diet feeding, there was no difference between mIGFREO/ApoE^−/−^ and ApoE^−/−^ in: (*A*) lipid deposition in aorta (representative images on left; *n* = 10, 21); scale bar denotes 5 mm; (*B*) circulating CD45^+^ leucocytes (*n* = 10, 8); (*C*) circulating CD45^+^CD11b^+^ myeloid cells (*n* = 10, 8); (*D*) circulating CD45^+^CD11b^+^Ly6G^hi^Ly6C^hi^ neutrophils (*n* = 10, 8); (*E*) circulating CD45^+^CD11b^+^Ly6G^−^Ly6C^+^ monocytes (*n* = 10, 8); (*F*) circulating CD45 ^+^ CD11b ^+^ Ly6G^−^Ly6C^hi^ ‘inflammatory’ monocytes (*n* = 10, 8); (*G*) circulating CD45^+^CD11b^+^Ly6G^−^Ly6C^lo^ ‘patrolling’ monocytes (*n* = 10, 8); (*H*) ratio of ‘inflammatory’ to ‘patrolling’ circulating monocytes (*n* = 10, 8); or (*I*) vascular permeability of Evans Blue dye in multiple vascular beds (*n* = 9, 6). Data expressed as mean (SEM). *n* denotes the number of mice per group. All statistical comparisons are made with unpaired Student’s *t*-tests.

Finally, to ascertain whether the kinase activity of the endothelial IGF-1R transgene reduced vascular permeability, circulating leucocyte abundance and atherogenesis *in vivo*, we crossed mIGFREO mice (with endothelium-specific transgenic expression of K1003R mutant human IGF-1R)^26^ with ApoE^−/−^ mice to generate mIGFREO/ApoE^−/−^. mIGFREO are identical to hIGFREO, except for modification of their IGF-1R transgene to the K1003R mutant we assessed *in vitro*. These mice were indistinguishable from their control littermates in terms of body mass before and after western diet feeding (see Supplementary material online, *Figure S11A* and *B*), fasting serum glucose (see Supplementary material online, *Figure S11C*), glucose tolerance (see Supplementary material online, *Figure S11D*) and insulin tolerance (see Supplementary material online, *Figure S11E*). However, when fed a western diet for 12 weeks, they were not protected against the development of atherosclerosis vs. littermate controls (*Figure 4A*). Moreover, they exhibited no reduction in total circulating leucocytes (*Figure 4B*), nor in subsets including: CD45^+^CD11b^+^ myeloid cells (*Figure 4C*), CD11b^+^Ly6G^+^Ly6C^hi^ neutrophils (*Figure 4D*), CD45^+^CD11b^+^Ly6G^−^Ly6C^+^ total monocytes (*Figure 4E*), CD11b^+^Ly6G^−^Ly6C^hi^ ‘inflammatory’ monocytes (*Figure 4F*), or CD11b^+^Ly6G^−^Ly6C^lo^ ‘patrolling’ monocytes (*Figure 4G*). The ratio of ‘inflammatory’ to ‘patrolling’ monocytes was also not significantly different (*Figure 4H*). Similarly, mIGFREO/ApoE^−/−^ mice were not protected against vascular leakage vs. littermate controls, in stark contrast with the phenotype of hIGFREO/ApoE^−/−^ (*Figure 4I*). These data corroborate the requirement for IGF-1R kinase activity for its modulation of endothelial permeability, circulating leucocytes, and atherogenesis.

## 4. Discussion

We demonstrate for the first time that increasing endothelial IGF-1R reduces atherosclerosis, modifies vascular endothelial junctions, reduces leakage between endothelial cells, and suppresses endothelial LDL-cholesterol uptake. This implies that endothelial IGF-1 receptors suppress multiple pathways that could lead to atherosclerosis. Moreover, this may be relevant to other disease processes caused by abnormal paracellular and/or transcellular transit across the vascular endothelium. Mechanistically, we prove that the kinase function of IGF-1R is required for these effects, indicating that they are not a structural property of the receptor, but instead represent signalling phenomena.

We have previously shown that ApoE^−/−^ mice with vascular endothelial over-expression of the human insulin receptor (referred to as hIRECO/ApoE^−/−^) develop increased atherosclerosis.^28^ These mice were generated and reared in an identical manner to hIGFREO/ApoE^−/−^, and so their opposing phenotypes are striking and suggest important differences in the signalling of these evolutionarily related receptors.^44^ Indeed, elegant studies from the group of Ronald Kahn have found important signalling differences in pre-adipocytes expressing chimeric insulin/IGF-1 receptors with exchange of their ligand binding and intracellular kinase domains.^45^ It is likely that multiple factors downstream of the activated IGF-1R are likely to be responsible for the phenotypes we have demonstrated. Our analyses suggest that WT IGF-1R suppresses nuclear localization of the transcription factor TAZ in endothelial cells, offering one potential explanation for the phenotype we observed. Indeed, YAP/TAZ silencing has been shown to reduce atherogenesis in mice,^43^ diminish endothelial VE-Cadherin turnover,^46^ and regulate clathrin-mediated endocytosis.^47^ In contrast to prior studies exploring the effects of insulin and IGF-1 on HUVEC,^48–52^ we did not find IGF-1R over-expression to increase the expression of the leucocyte adhesion molecules VCAM-1 and ICAM-1. Those studies implicated downstream signalling via p38 and ERK,^49,52^ which we also did not observe. This may reflect differing downstream signalling induced by a modest and sustained signalling stimulus in our model vs. the intense and short-lived stimulus arising from exposure to large concentrations of IGF-1 or insulin. Further mechanistic studies are needed to understand these differences and may help to guide therapeutic translation.

It is important to note that whilst endothelial IGF-1R over-expression suppresses atherogenesis, administration of IGF-1 ligand is unlikely to represent a means to achieve clinical translation. IGF-1 induced acromegaly-like side effects in clinical trials as a diabetes therapy,^53^ and more importantly is a mechanistically distinct intervention from IGF-1R over-expression. Much more work is needed to define how IGF-1R influences endothelial TAZ localization, but understanding this novel observation provides a prospective path to translating our findings to clinical benefit in modulating atherogenesis and other diseases linked to abnormal vascular barrier function. Although YAP/TAZ inhibitors are being developed,^54^ these are also likely to have wide-ranging systemic effects, and so it is likely that defining endothelial-specific targets arising from our findings may lead to more refined therapies.

Our work extends the findings of other groups who noted IGF-1R knockout to increase endothelial leakage.^21,22^ These groups reached differing conclusions regarding the underlying mechanisms, noting altered expression of multiple junctional proteins,^22^ or altered phosphorylation of VE-Cadherin.^21^ We concur with some of their findings in regard to IGF-1R diminishing paracellular permeability, yet we note altered junctional abundance on immunofluorescence, rather than expression or modification of the junctional proteins claudin-5 and VE-Cadherin. Notably, diminution of YAP/TAZ signalling reduces VE-Cadherin turnover,^46^ offering a potential explanation for our findings. There are also some parallels between our data and studies of IGF-1 regulation of gut epithelia, with multiple studies showing IGF-1 induced expression of epithelial claudins and reduction in paracellular leakage.^55^ However, whilst paracellular vascular permeability is potentially relevant to atherogenesis, much of the LDL-cholesterol that forms atherosclerotic plaque is actively transported across endothelial cells. Our finding that increased IGF-1R expression reduces endothelial LDL uptake *in vitro* and *in vivo* is therefore a particularly important and novel observation. This adds to the recent elegant studies of Yang *et al.*^41^ who identified brain endothelial Igf1r expression as being robustly associated with transcellular transport activity. Notably, Igf1r expression is enriched in brain endothelial cells, vs. those from other organs, and so our data imply that IGF-1R could have a causal role in the association noted by Yang *et al.*, although this requires further exploration. Whether downstream modulation of YAP/TAZ is relevant to their data is unclear and will be an important direction for future neurovascular research. It will also be important for future studies to corroborate our findings in human arterial endothelial cells, given their relevance to atherosclerosis.

Our data are particularly relevant to the phenomenon of atherosclerosis and suggest that diminished vascular IGF-1 signalling promotes atherogenesis, at least in part, via increased paracellular and transcellular vascular permeability. However, there may be wider implications for other diseases associated with abnormal vascular permeability, ranging from generalized diseases like sepsis or cancer to organ-specific diseases like dementia. Further exploration is needed of the downstream mechanisms by which IGF-1, but not insulin, receptor signalling promotes these phenomena in endothelial cells. In conjunction with our findings, these data could have wide-ranging implications for our understanding of vascular biology and lead to novel disease therapeutics to normalize multiple aspects of vascular barrier function.

Translational perspective Atherosclerosis is initiated by circulating cholesterol-rich lipoproteins passing across the arterial endothelial barrier. We show that increasing endothelial over-expression of insulin-like growth factor-1 receptors (IGF-1Rs) reduces leakage between and across endothelial cells, which is associated with reduced atherogenesis. The signalling function of IGF-1R is required for this process, which may explain some of the anti-atherosclerotic properties of its ligand IGF-1. Our data suggest that augmenting vascular IGF-1 signalling has the potential to augment vascular barrier function, which has the potential not only to reduce atherogenesis, but also many other diseases associated with vascular leakage.

## Supplementary Material

cvaf055_Supplementary_Data

## Data Availability

Raw data are available on request from the corresponding author. RNA-seq data have been deposited under accession ID E-MTAB-14458 at ArrayExpress (https://www.ebi.ac.uk/biostudies/arrayexpress).
