# Supplementary material for: Endothelial insulin-like growth factor-1 signalling regulates vascular barrier function and atherogenesis
Source: Cardiovasc Res. 2025 Apr 2;121(7):1108–20. doi: 10.1093/cvr/cvaf055 (PMC12236071; doi:10.1093/cvr/cvaf055)
Supplement: cvaf055_Supplementary_Data [file cvaf055_supplementary_data.zip › hIGFREO-ApoE CVR R2 supplementary material.docx]

**Endothelial insulin-like growth factor-1 signaling regulates vascular barrier function and atherogenesis**

Michael Drozd PhD^1^*, Alexander-Francisco Bruns PhD^1^*, Nadira Y Yuldasheva PhD^1^*, Azhar Maqbool PhD^1^, Hema Viswambharan PhD^1^, Anna Skromna MSc^1^, Natallia Makava MSc^1^, Chew W Cheng PhD^1^, Piruthivi Sukumar PhD^1^, Lauren Eades PhD^1^, Andrew MN Walker PhD^1^, Kathryn J Griffin PhD^1^, Stacey Galloway BSc^1^, Nicole T Watt PhD^1^, Natalie Haywood PhD^1^, Victoria Palin MBChB^1^, Nele Warmke PhD^1^, Helen Imrie PhD^1^, Katherine Bridge PhD^1^, David J Beech PhD^1^, Stephen B Wheatcroft PhD^1^, Mark T Kearney MD^1^, Richard M Cubbon PhD^1^.

^1^ Leeds Institute of Cardiovascular and Metabolic Medicine, LIGHT Laboratories, Clarendon Way, University of Leeds, Leeds, LS2 9JT, United Kingdom.

* denotes equal contribution

**Address for correspondence and material requests:**

Dr Richard Cubbon

Leeds Institute of Cardiovascular and Metabolic Medicine,

LIGHT Laboratories 7.04, Clarendon Way,

University of Leeds, Leeds, LS2 9JT,

United Kingdom.

E-mail: [r.cubbon@leeds.ac.uk](mailto:r.cubbon@leeds.ac.uk)

**Supplemental material description**

**Supplemental Figure 1: hIGFREO/ApoE^-/-^ mice develop normally and have appropriate endothelial expression of the human IGF-1R transgene. a)** No difference in body mass of hIGFREO/ApoE^-/-^ compared to ApoE^-/-^ littermates between onset of western diet feeding (WDF) at 8 weeks of age, and completion of western diet feeding at 20 weeks of age (*n*=17,20,12,19); **b)** Increased expression of total (human and murine) IGF-1R protein in endothelial cells from hIGFREO/ApoE^-/-^ versus ApoE^-/-^ littermates (*n*=6,20); **c)** Human IGF-1R mRNA was not expressed in non-endothelial cells of hIGFREO/ApoE^-/-^ or in endothelial cells from ApoE^-/-^ littermates (*n*=9,11,5); **d)** Human IGF-1R was barely detectable in CD11b^+^ myeloid cells from hIGFREO/ApoE^-/-^ (*n*=3,3,3). Data expressed as mean (SEM) * denotes P<0.05; *n* denotes number of mice per group; all statistical comparisons are made with unpaired Student t-tests.

**Supplemental Figure 2: Expression of leukocyte adhesion molecules is similar in the aorta and pulmonary endothelial cells of hIGFREO/ApoE^-/-^ and ApoE^-/-^ littermates after 12 weeks of western diet feeding. a)** Aortic VCAM1 mRNA (*n*=9,8); **b**) Aortic VCAM1 protein (note that a blank lane is present between ApoE^-/-^ and hIGFREO/ApoE^-/-^ lanes in representative blot; *n*=8,7); **c**) Aortic ICAM1 mRNA (*n*=9,8); **d**) Aortic ICAM1 protein (note that a blank lane is present between ApoE^-/-^ and hIGFREO/ApoE^-/-^ lanes in representative blot; *n*=7,7); **e**) Pulmonary endothelial cell VCAM1 protein (*n*=8,17); **f**) Pulmonary endothelial cell ICAM1 protein (*n*=8,17). Data expressed as mean (SEM); *n* denotes number of mice per group; all statistical comparisons are made with unpaired Student t-tests.

**Supplemental Figure 3: The metabolic profile of hIGFREO/ApoE^-/-^ is comparable to ApoE^-/-^ controls.** After 12 weeks of feeding, there was no difference between hIGFREO/ApoE^-/-^ and ApoE^-/-^ in: **a)** Fasting glucose (*n*=7,8); **b)** Glucose tolerance (*n*=5,5); **c)** Insulin tolerance (*n*=5,5); **d)** Fasting serum insulin (*n*=12,13); **e)** Serum IGF-1 (*n*=10,10); **f)** Serum triglycerides (*n*=11,17); or, **g)** Total cholesterol (*n*=11,17) in hIGFREO/ApoE^-/-^ compared to ApoE^-/-^ littermates. Data expressed as mean (SEM); *n* denotes number of mice per group; all statistical comparisons are made with unpaired Student t-tests.

**Supplemental Figure 4: Systolic blood pressure and aortic vasomotor responses are unchanged in hIGFREO/ApoE^-/-^.** After 12 weeks of feeding, there was no difference between hIGFREO/ApoE^-/-^ and ApoE^-/-^ in: **a)** Systolic blood pressure (*n*=12,19); **b)** Acetylcholine-induced aortic relaxation (*n*=7,14); **c)** Sodium nitroprusside (SNP)-induced aortic relaxation (*n*=7,14); **d)** Phenylephrine-induced aortic constriction (*n*=7,14); **e,f)** Aortic constriction to the non-selective nitric oxide synthase (NOS) inhibitor L-NMMA, after phenylephrine pre-constriction (*n*=7,14). Data expressed as mean (SEM); *n* denotes number of mice per group; statistical comparisons for panels a and f are made with unpaired Student t-tests, and repeated measures ANOVA for panels b to e.

**Supplemental Figure 5: Vascular oxidative stress, NADPH oxidase (NOX) isoform -2 and -4 expression, and eNOS expression and phosphorylation were similar in hIGFREO/ApoE^-/-^ versus ApoE^-/-^ littermates after 12 weeks western diet feeding. a)** MnTMPyP-induced blunting of acetylcholine-induced aortic relaxation (*n*=4,3). **b**) Aortic NOX2 and NOX4 expression (note that a blank lane is present between ApoE^-/-^ and hIGFREO/ApoE^-/-^ lanes in representative blot; *n*=11,12, 10,13). **c)** Pulmonary endothelial cell NOX2 and NOX4 expression (*n*=7,15, 7,16). **d)** Basal aortic S1177-phospho-eNOS to total eNOS ratio (*n*=10,10); **e)** Insulin stimulated NOS activity (*n*=9,8); **f)** IGF-1 stimulated NOS activity (*n*=5,5). Data expressed as mean (SEM); *n* denotes number of mice per group; statistical comparisons for panels a-b are made with repeated measures ANOVA, and unpaired Student t-tests for panels c-f.

**Supplemental Figure 6: Circulating, but not bone marrow, leukocyte populations are reduced in hIGFREO/ApoE^-/-^. a)** Circulating CD45^+^CD11b^+^ myeloid cells (*n*=15,15); **b)** Circulating CD45^+^CD11b^+^Ly6G^-^Ly6C^+^ monocytes (*n*=15,15); **c)** Circulating CD45^+^CD11b^+^Ly6G^-^Ly6C^hi^ ‘inflammatory’ monocytes (*n*=15,15); **d)** Circulating CD45^+^CD11b^+^Ly6G^-^Ly6C^lo^ ‘patrolling’ monocytes (*n*=15,15); **e)** Circulating CD45^+^CD11b^+^Ly6G^hi^Ly6C^hi^ neutrophils (*n*=15,15); **f)** Ratio of ‘inflammatory’ to ‘patrolling’ circulating monocytes (*n*=15,15); **g)** Bone marrow CD45^+^CD11b^+^ myeloid cells (*n*=8,13); **h)** Bone marrow CD45^+^CD11b^+^Ly6G^-^Ly6C^+^ monocytes (*n*=8,13); **i)** Bone marrow CD45^+^CD11b^+^Ly6G^hi^Ly6C^hi^ neutrophils (*n*=8,13); **j)** Bone marrow Lin^-^Sca-1^+^c-Kit^+^ hematopoietic stem cells (*n*=8,13). Data expressed as mean (SEM); * denotes P<0.05; *n* denotes number of mice per group; all statistical comparisons are made with unpaired Student t-tests.

**Supplemental Figure 7: Transplantation of hIGFREO/ApoE^-/-^ donor bone marrow in to ApoE^-/-^ recipients does not influence circulating leukocyte abundance or the development of atherosclerosis. a)** Schema of bone marrow transplantation experiments. Transplantation of hIGFREO/ApoE^-/-^ donor bone marrow, versus ApoE^-/-^ donor bone marrow, in to ApoE^-/-^ recipients does not alter: **b)** Circulating total CD45^+^ leukocytes (*n*=10,9); **c)** Circulating myeloid cells (*n*=10,9); **d)** Ratio of ‘inflammatory’ to ‘patrolling’ circulating monocytes (*n*=10,9); **e)** Bone marrow total CD45^+^ leukocytes (*n*=10,8); **f)** Bone marrow myeloid cells (*n*=10,8); **g)** Bone marrow Lin^-^Sca-1^+^c-Kit^+^ hematopoietic stem cells (*n*=10,8); **h)** Total aortic atherosclerotic plaque area defined by Oil-Red O staining (*n*=13,14); scale bar denotes 5 mm. Data expressed as mean (SEM); *n* denotes number of mice per group; all statistical comparisons are made with unpaired Student t-tests.

**Supplemental Figure 8: Overexpression of wildtype IGF-1R in human endothelial cells alters the localization, but not expression or post-translational modification, of junction proteins. a**) Immunoblotting of human umbilical vein endothelial cells (HUVEC) overexpressing wildtype or K1003R IGF-1R reveals neither alters the expression of claudin-5, CD31, occludin or VE-Cadherin (*n*=5,5,5,5). **b**) Representative images of HUVEC claudin-5 with either wildtype or K1003R IGF1R overexpression (Claudin-5 – red, DAPI – blue; scale bar denotes 50 μm). HUVEC overexpressing wildtype or K1003R IGF-1R exhibit no difference in VE-Cadherin Y731 (*n*=3,3). **c**) Claudin-5 junctional area is increased in HUVEC overexpressing wildtype, but not K1003R (*n*=12,12,12,12). Data expressed as mean (SEM); * denotes P<0.05; *n* denotes number of donors per group; all statistical comparisons are made with unpaired Student t-tests.

**Supplemental Figure 9: Overexpression of wildtype, but not K1003R, IGF-1R in human endothelial cells increases colocalization of administered LDL cholesterol with clathrin, but not caveolin-1.** Representative confocal images of ApoB (green) and clathrin heavy chain (red) or caveolin-1 (red) immunofluorescence in human umbilical vein endothelial cells expressing either wildtype or K1003R IGF-1R 30 minutes after exposure to human LDL-cholesterol. Green denotes ApoB fluorescence and red denotes caveolin-1 or clathrin heavy chain fluorescence. Expression of wildtype, but not K1003R, IGF-1R increased colocalization of ApoB with clathrin; no difference was noted for colocalization with caveolin-1. Data expressed as mean (SEM); * denotes P<0.05; *n=3* per group; all statistical comparisons are made with unpaired Student t-tests.

**Supplemental Figure 10: Overexpression of wildtype or K1003R IGF-1R does not influence inflammatory signaling in human endothelial cells. a)** Representative immunoblots accompanying panel b, showing expression of IGF-1R, phospho-IGF-1R (Y1135), Akt, phospho-Akt (S473), JNK, phospho-JNK (T183/Y185), ERK1/2, phospho-ERK1/2 (T202/Y204), p38, phospho-p38 (T180/Y182) and HSP90 loading control. **b)** Quantification of immunoblotting reveals a nominal increase in phospho-IGF-1R in human umbilical vein endothelial cells expressing wildtype IGF-1R, but no impact of wildtype or K1003R IGF-1R overexpression on Akt, JNK, ERK or p38 phosphorylation. **c)** NF-kB activity is not altered in human umbilical vein endothelial cells expressing wildtype or K1003R IGF-1R, although stimulation with tumor necrosis factor-alpha 10ng/mL (TNF-α) for 4h induces robust activation. **d)** Cell surface expression of VCAM-1 and ICAM-1 is not altered in human umbilical vein endothelial cells expressing wildtype or K1003R IGF-1R, although stimulation with TNF-α 10ng/mL for 4h induces robust induction of surface expression, defined as geometric mean fluorescence intensity (MFI) and percentage of cells with expression. Data expressed as mean (SEM); *n=3-4* per group; all statistical comparisons are made with unpaired Student t-tests.

**Supplemental Figure 11: The metabolic profile of mIGFREO/ApoE^-/-^ is comparable to ApoE^-/-^ controls. a)** Experimental schema; **b**) Body mass at onset of western diet at 8 weeks of age, and completion of western diet feeding at 20 weeks of age (*n*=29,26,26,27); **c)** Fasting glucose (*n*=13,29); **d)** Glucose tolerance (*n*=13,29); **e)** Insulin tolerance (*n*=16,11). Data expressed as mean (SEM); *n* denotes number of mice per group; all statistical comparisons are made with unpaired Student t-tests.

**Supplemental Figure 12: Flow cytometry gating strategies.** Representative images of gating strategies used to define blood and bone marrow-resident leukocytes (upper panel), bone marrow-resident haematopoietic stem cells (middle panel), and human umbilical vein endothelial cell surface expression of VCAM-1 and ICAM1 (lower panel; TNF-a denotes tumor necrosis factor alpha).

**Supplemental Data 1:** RNA-sequencing comparison of human umbilical endothelial cells overexpressing WT IGF-1R versus K1003R IGF-1R

**Supplemental Data 2:** g:Profiler functional enrichment analysis of 48 differentially expressed genes identified in human umbilical endothelial cells overexpressing WT IGF-1R versus K1003R IGF-1R (presented in Table 1).
