# Supplementary figures and images for: Endothelial insulin-like growth factor-1 signalling regulates vascular barrier function and atherogenesis
Source: Cardiovasc Res. 2025 Apr 2;121(7):1108–20. doi: 10.1093/cvr/cvaf055 (PMC12236071; doi:10.1093/cvr/cvaf055)

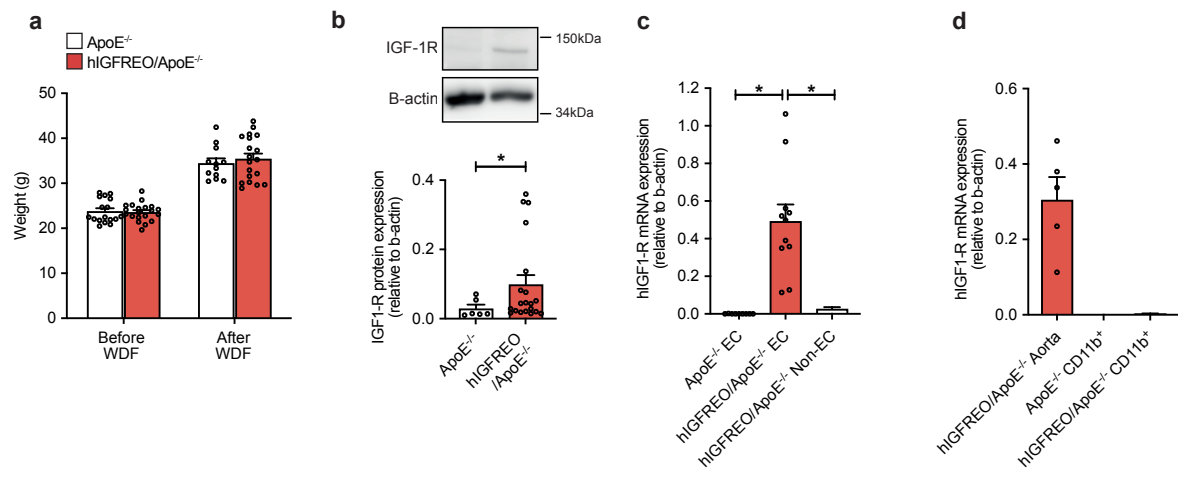

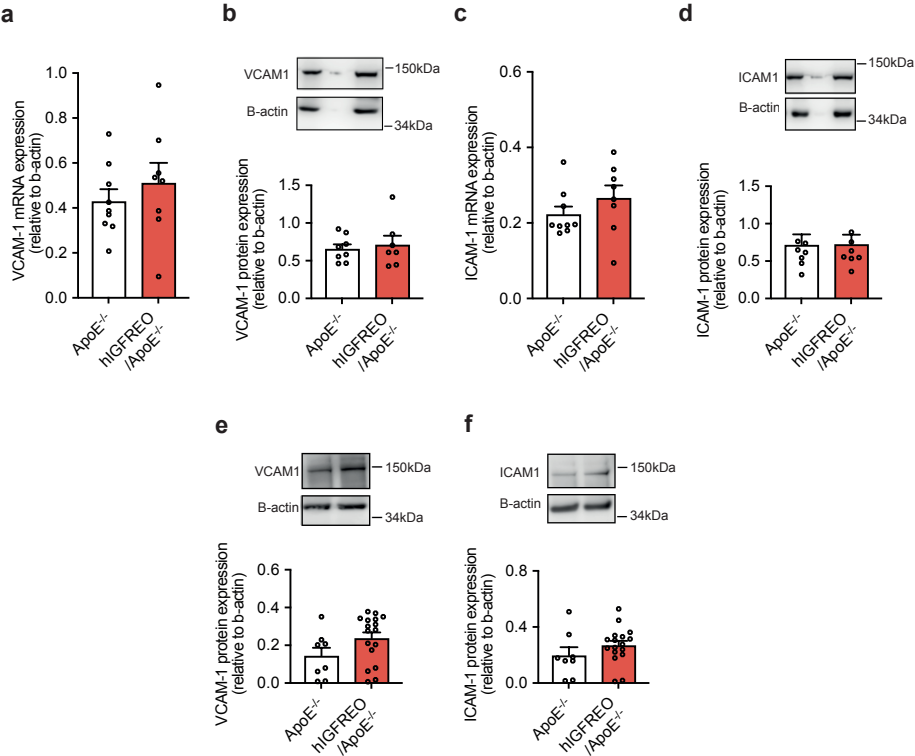

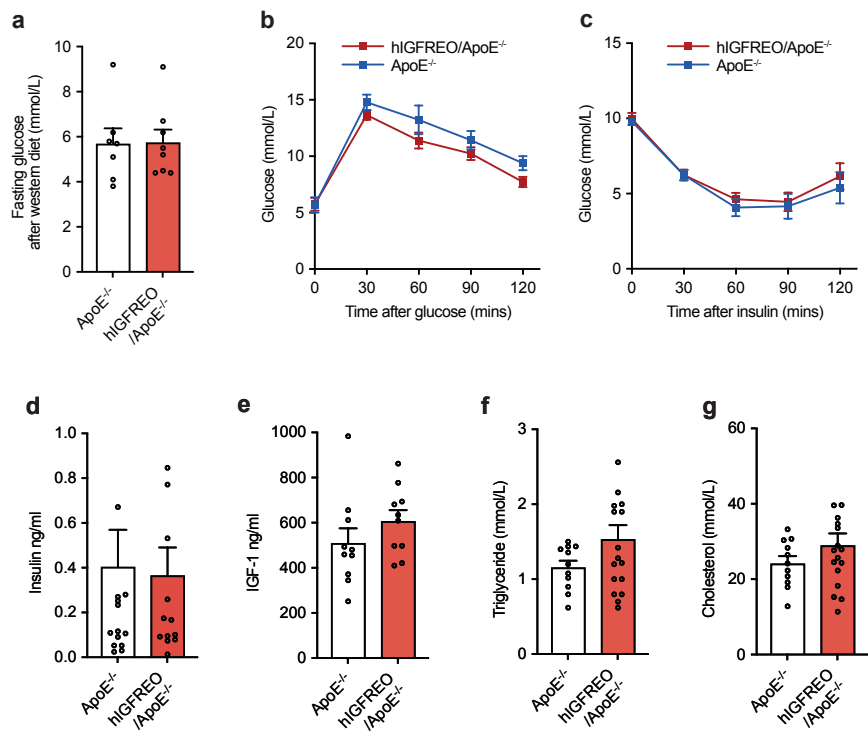

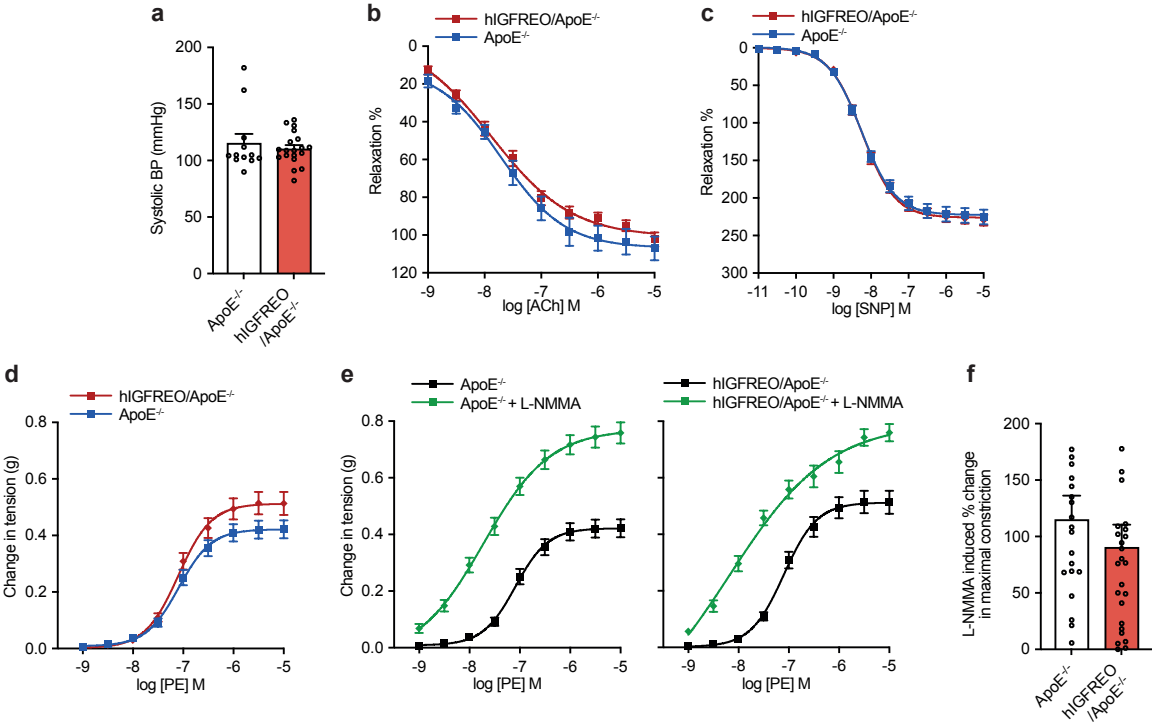

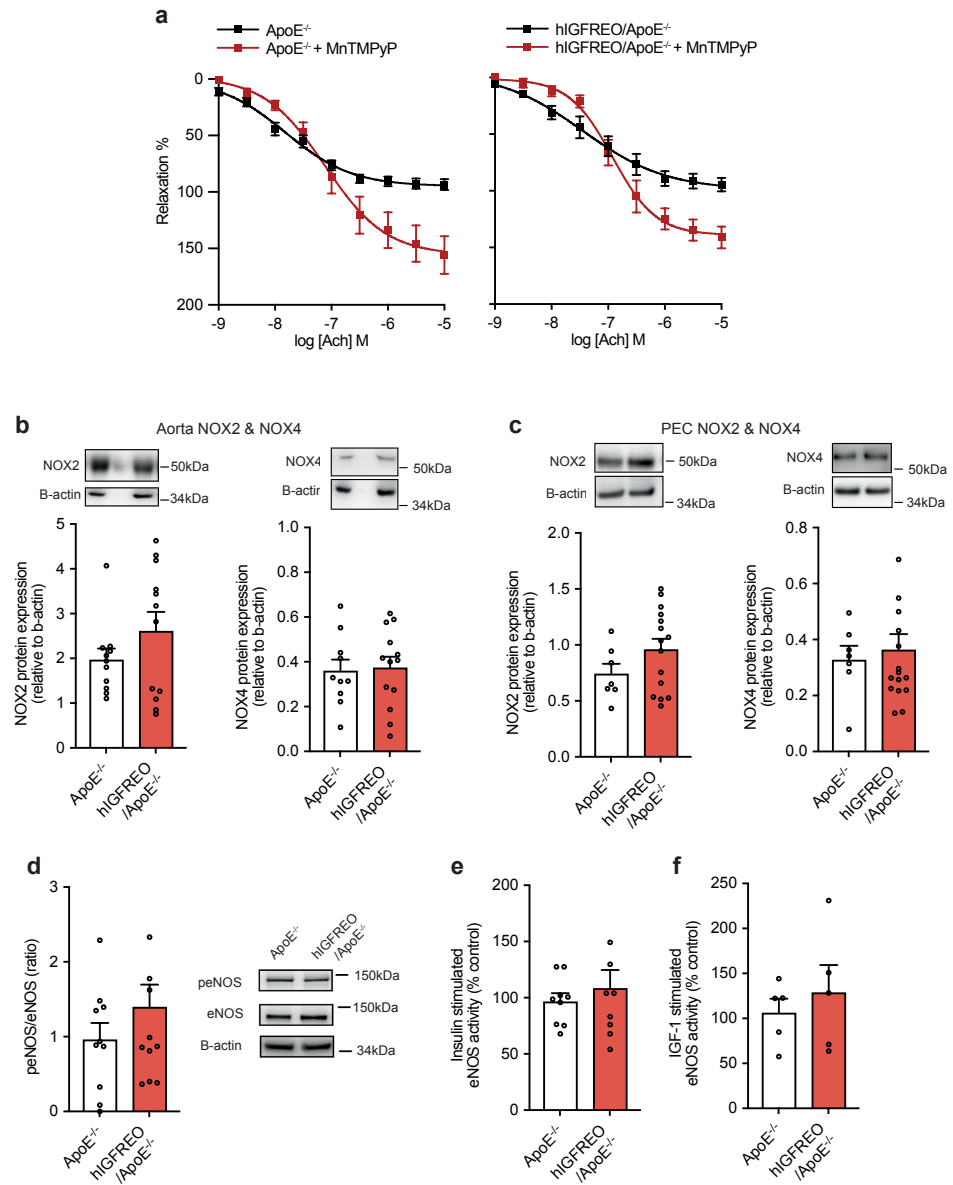

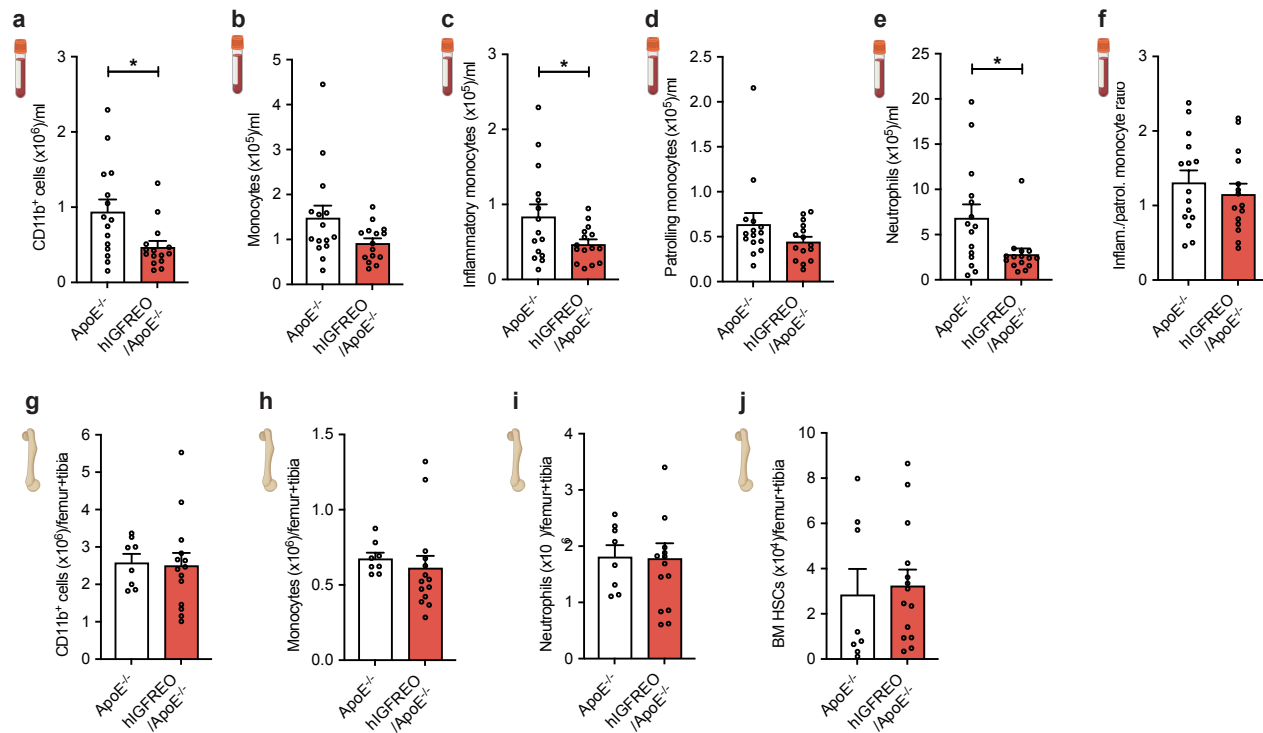

# Supplementary 7

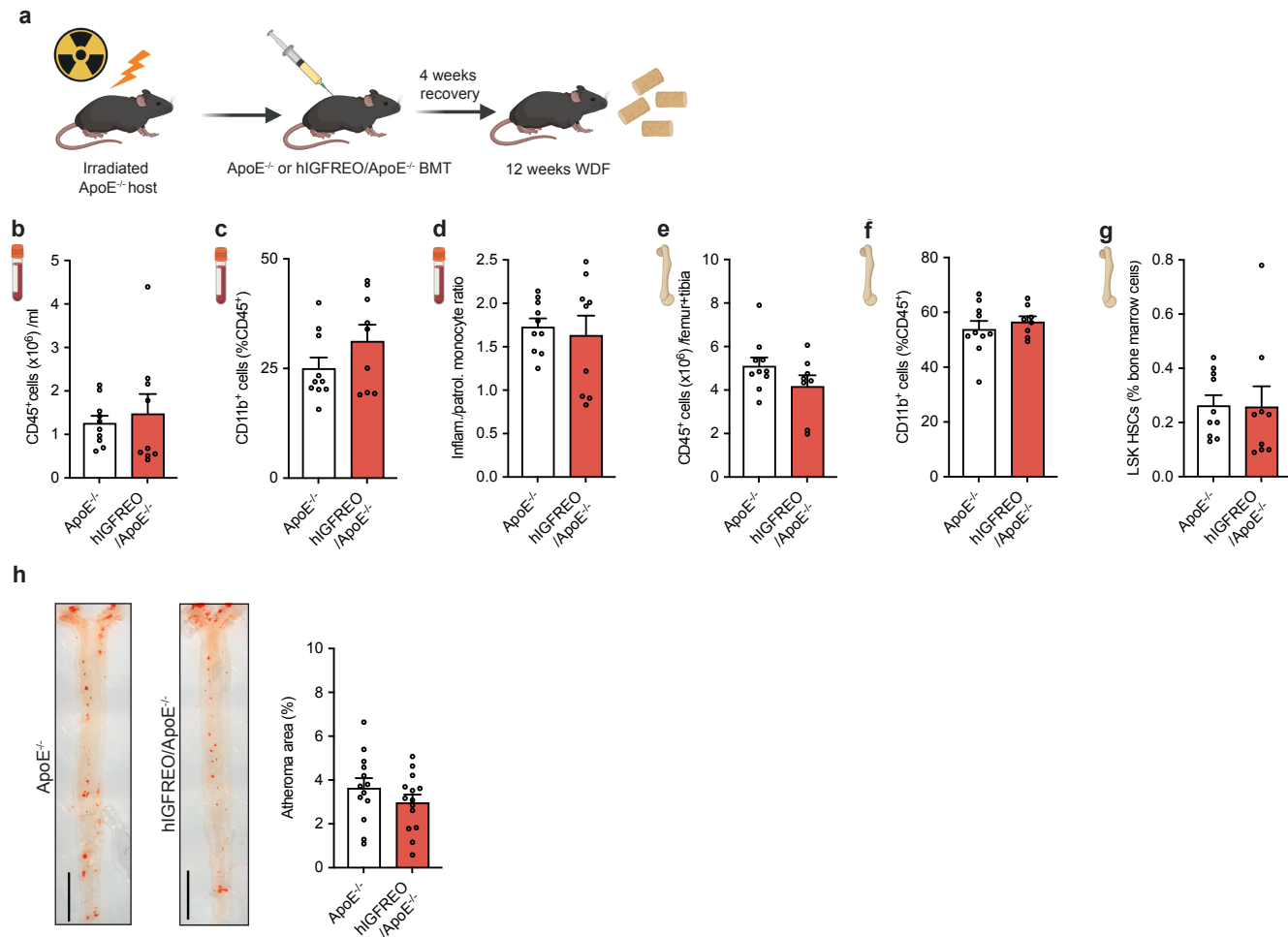

**a**

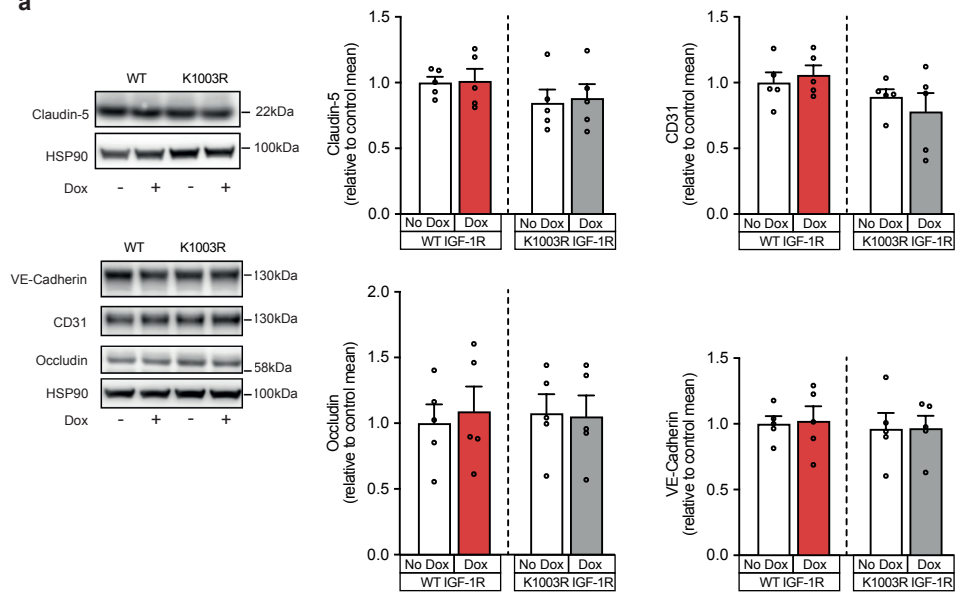

**b**

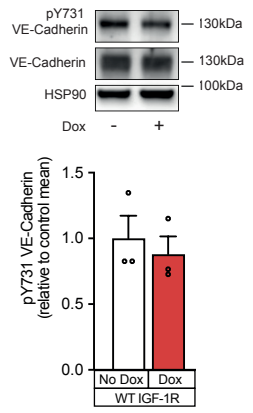

**c**

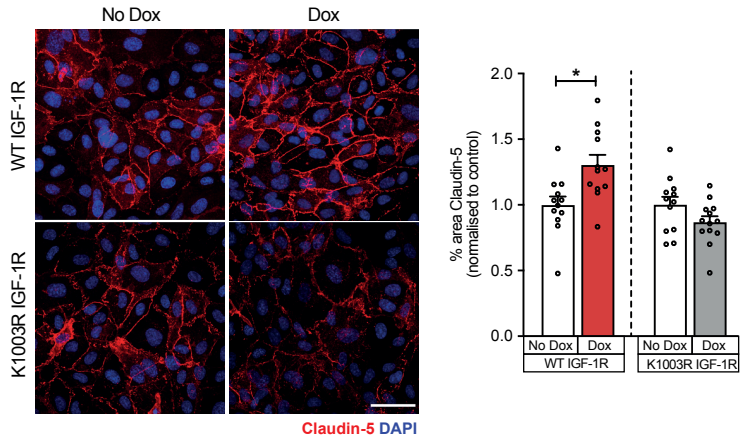

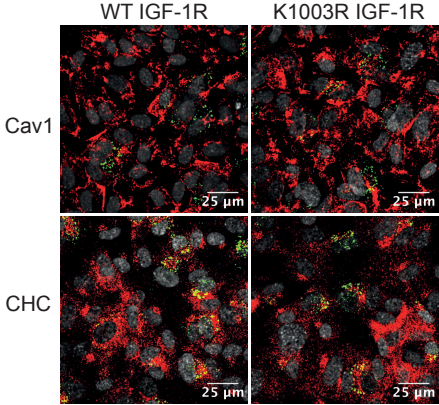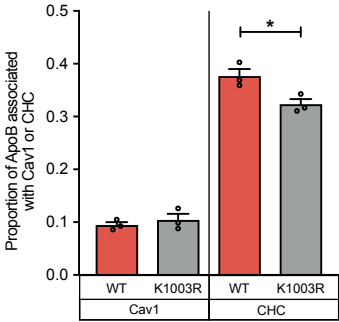

**a**

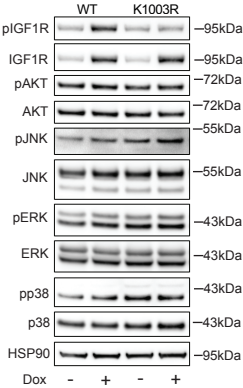

**b**

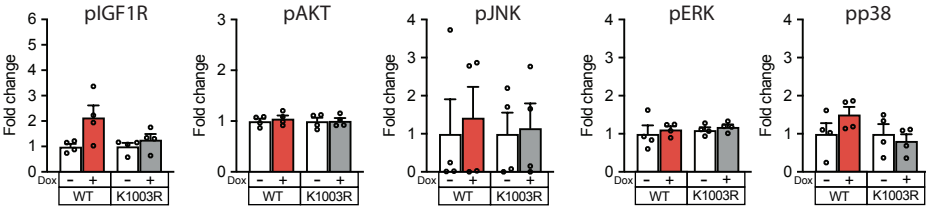

**c**

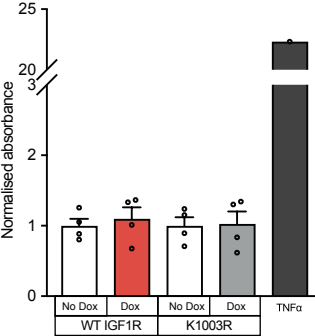

**d**

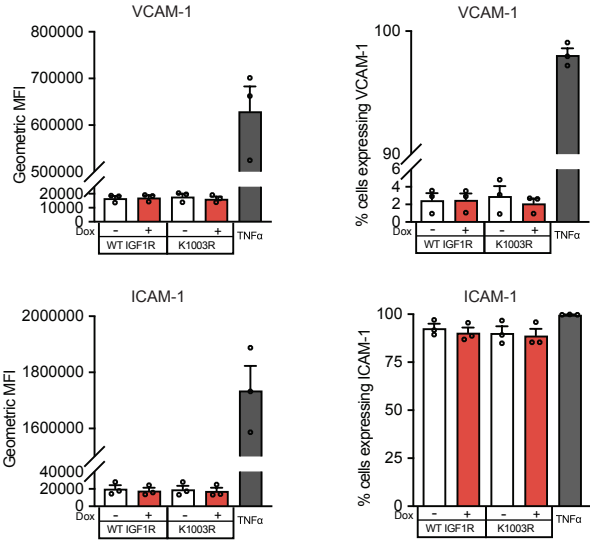

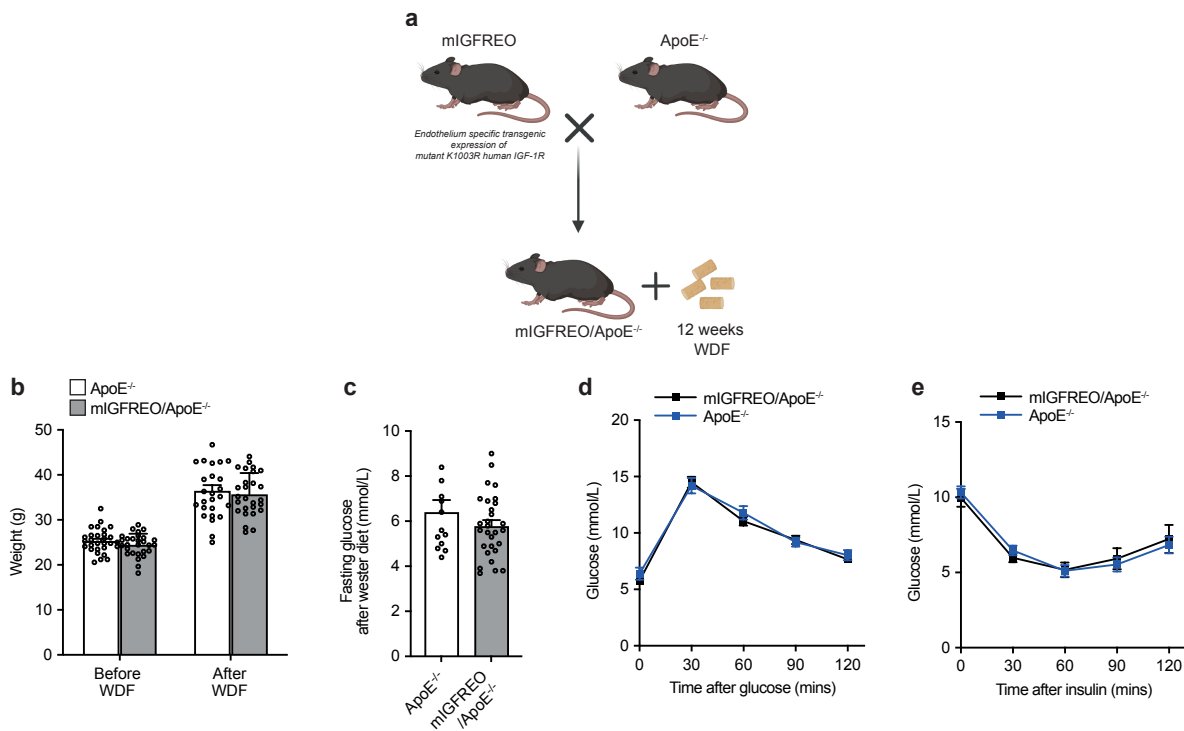

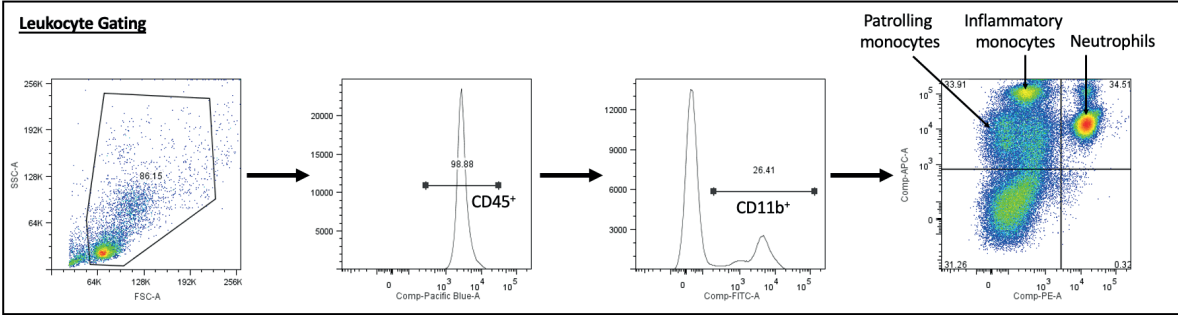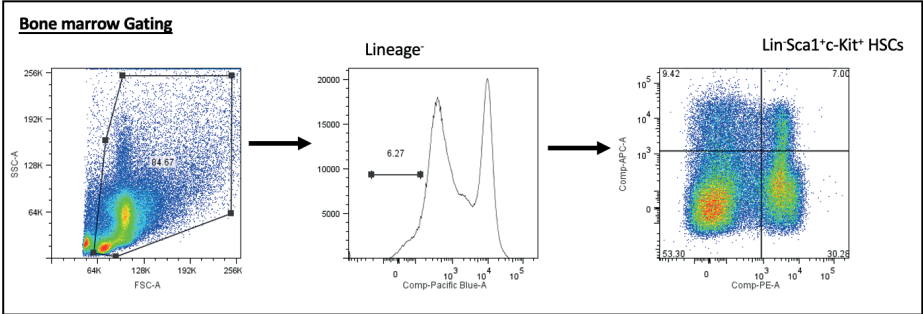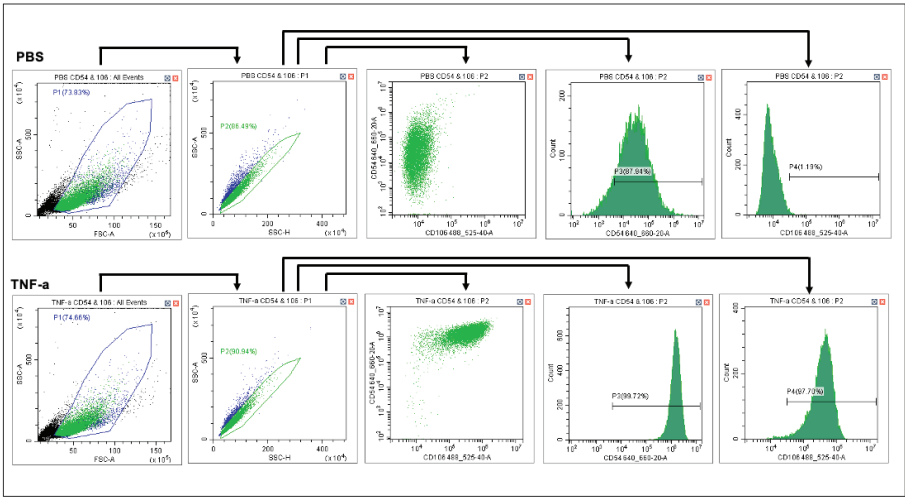

Supplement: cvaf055_Supplementary_Data [file cvaf055_supplementary_data.zip › SupplementaryFiguresR2.pdf]
